# Analysis of genetic variants in myeloproliferative neoplasms using a 22-gene next-generation sequencing panel

**DOI:** 10.1186/s12920-021-01145-0

**Published:** 2022-01-15

**Authors:** Jaymi Tan, Yock Ping Chow, Norziha Zainul Abidin, Kian Meng Chang, Veena Selvaratnam, Nor Rafeah Tumian, Yang Ming Poh, Abhi Veerakumarasivam, Michael Arthur Laffan, Chieh Lee Wong

**Affiliations:** 1 Department of Biological Sciences, School of Medical and Life Sciences, Sunway University, Petaling Jaya, Selangor Malaysia; 2 Clinical Research Centre, Sunway Medical Centre, Petaling Jaya, Selangor Darul Ehsan Malaysia; 3 Molecular Diagnostics Laboratory, Sunway Medical Centre, Petaling Jaya, Selangor Darul Ehsan Malaysia; 4 Haematology Unit, Department of Medicine, Sunway Medical Centre, Petaling Jaya, Selangor Darul Ehsan Malaysia; 5 Haematology Department, Ampang Hospital, Kuala Lumpur, Malaysia; 6 Haematology Unit, Department of Medicine, Universiti Kebangsaan Malaysia Medical Centre, Kuala Lumpur, Malaysia; 7 School of Data Sciences, Perdana University, Serdang, Selangor Malaysia; 8 Centre for Haematology, Hammersmith Hospital, London, UK; 9 Faculty of Medicine, Imperial College London, London, UK

**Keywords:** Myeloproliferative neoplasm, Next-generation sequencing, Gene, Mutation, Variant, Bioinformatics, disease management

## Abstract

**Background:**

The Philadelphia (Ph)-negative myeloproliferative neoplasms (MPNs), namely essential thrombocythaemia (ET), polycythaemia vera (PV) and primary myelofibrosis (PMF), are a group of chronic clonal haematopoietic disorders that have the propensity to advance into bone marrow failure or acute myeloid leukaemia; often resulting in fatality. Although driver mutations have been identified in these MPNs, subtype-specific markers of the disease have yet to be discovered. Next-generation sequencing (NGS) technology can potentially improve the clinical management of MPNs by allowing for the simultaneous screening of many disease-associated genes.

**Methods:**

The performance of a custom, in-house designed 22-gene NGS panel was technically validated using reference standards across two independent replicate runs. The panel was subsequently used to screen a total of 10 clinical MPN samples (ET n = 3, PV n = 3, PMF n = 4). The resulting NGS data was then analysed via a bioinformatics pipeline.

**Results:**

The custom NGS panel had a detection limit of 1% variant allele frequency (VAF). A total of 20 unique variants with VAFs above 5% (4 of which were putatively novel variants with potential biological significance) and one pathogenic variant with a VAF of between 1 and 5% were identified across all of the clinical MPN samples. All single nucleotide variants with VAFs ≥ 15% were confirmed via Sanger sequencing.

**Conclusions:**

The high fidelity of the NGS analysis and the identification of known and novel variants in this study cohort support its potential clinical utility in the management of MPNs. However, further optimisation is needed to avoid false negatives in regions with low sequencing coverage, especially for the detection of driver mutations in *MPL*.

**Supplementary Information:**

The online version contains supplementary material available at 10.1186/s12920-021-01145-0.

## Background

Myeloproliferative neoplasms (MPNs) are a group of chronic clonal haematopoietic disorders. The MPN subtypes include the Philadelphia (Ph) chromosome-positive chronic myeloid leukaemia, as well as the Ph-negative MPNs such as essential thrombocythaemia (ET) (characterised by an overproduction of platelets), polycythaemia vera (PV) (characterised by an overproduction of red blood cells), and primary myelofibrosis (PMF) (characterised by progressive bone marrow fibrosis, and is further subdivided into an early, prefibrotic stage (pre-PMF) and a late, overt fibrotic stage (overt-PMF)). Among the three, ET is the most indolent, whereas PMF is associated with the highest symptom burden and worst prognosis. Common causes of morbidity and mortality include thromboembolic and/or haemorrhagic complications, as well as disease progression to myelofibrosis (MF) and/or transformation to acute myeloid leukaemia (AML), all of which vary in frequencies between MPN subtypes [1]. To date, allogenic stem cell transplantation remains the only curative option for MPNs but it is often not considered due to age-related co-morbidities and high transplant-associated mortality rates. Hence, available treatment options such as phlebotomy, aspirin, hydroxyurea, anagrelide, pegylated interferons, and JAK inhibitors are primarily aimed at reducing the risk of disease complications, disease progression, and malignant transformation [2, 3].

The discovery of driver mutations in the *JAK2*, *CALR* and *MPL* genes contributed towards the improved accuracy of MPN diagnostics. The *JAK2* gene encodes the JAK2 non-receptor tyrosine kinase associated with several receptors which are critical for normal myelopoiesis, including the erythropoietin, thrombopoietin, and granulocyte colony-stimulating factor receptors; the *MPL* gene encodes the thrombopoietin receptor; while the *CALR* gene encodes the chaperone protein calreticulin. Driver mutations in these genes (*JAK2* p.V617F or exon 12 mutations, *CALR* exon 9 insertions and deletions, and *MPL* W515 mutations) lead to the constitutive activation of associated receptors and the subsequent amplification of downstream signalling pathways, which result in the aberrant cell proliferation in MPN. However, these driver mutations are not MPN subtype-specific, and can also be found in other myeloid diseases such as myelodysplastic syndromes (MDS), myelodysplastic/myeloproliferative neoplasms (MDS/MPN) and AML [4, 5]. In addition, there are also MPN cases that are triple-negative (TN) for all the driver mutations.

The management of MPNs requires an integrated, multimodal approach that includes the evaluation of clinical features, peripheral blood smear, bone marrow morphology, immunophenotype, cytogenetics, as well as molecular genetics, as recommended in the 2016 World Health Organisation (WHO) classification guidelines [4]. As MPNs are chronic in nature, cases are often asymptomatic upon diagnosis and discovered incidentally through physical examination or abnormal blood counts [4]. Typically, the diagnostic procedure includes molecular tests for MPN driver mutations, as well as the morphological analysis of the peripheral blood smear and bone marrow aspirate/trephine biopsy; the findings of which are correlated with the results of the full blood count. However, morphological features such as the degree of bone marrow fibrosis used for disease diagnosis are subjective in nature and thus have high inter-observer variability [6–8]. Therapeutic decisions are then made based on the patient’s mutational landscape, disease burden, as well as disease prognosis. Nevertheless, the significant genotypic and phenotypic heterogeneity that exist between MPN subtypes and other myeloid disorders remains a challenge towards effective management of MPNs.

Rapid advancements in gene sequencing technology in the last decade have led to the discovery of other MPN-associated genetic aberrations, such as mutations in *ASXL1*, *EZH2*, *TET2*, *IDH1*, *IDH2*, *SRSF2* and *SF3B1*, contributing towards improved disease prognostication. In a novel prognostic model developed by Grinfeld*, *et al*.* [9], patients with MPN were stratified into eight groups by combining genomic data with traditional laboratory and clinical findings. Compared to current prognostic models, the model was superior in performance and was able to better define disease outcomes, especially within the “intermediate-risk” categories of the current prognostic models [9]. However, more comprehensive genetic profiling of patients using next-generation sequencing (NGS) technology is required before such a model can be implemented in clinics worldwide.

In this study, we report on the validation of a custom NGS panel which targets 22 MPN-associated genes (*ABL1*, *ASXL1*, *CALR*, *CBL*, *CEBPA*, *CSF3R*, *DNMT3A*, *EZH2*, *FLT3*, *IDH1*, *IDH2*, *JAK2*, *KIT*, *MPL*, *NPM1*, *PDGFRA*, *RUNX1*, *SF3B1*, *SRSF2*, *TET2*, *TP53*, and *U2AF1*) by using a set of reference standards and a small cohort of clinical MPN samples.

## Materials and methods

### Ethics statement

A multicentre study was conducted across three institutions in Malaysia, namely Sunway Medical Centre, Ampang Hospital, and Universiti Kebangsaan Malaysia Medical Centre. Ethics approval for this study was obtained from the Sunway Medical Centre Independent Research Ethics Committee (SREC 008/2018/FR), Universiti Kebangsaan Malaysia Medical Centre Research Ethics Committee (UKM FPR.4/244/FF-2018-420), Medical Research & Ethics Committee Ministry of Health Malaysia (MREC 8KKM/NIHSEC/P-19-99), and Sunway University Research Ethics Committee (PGSUREC 2019/010).

### Custom NGS panel

An Ampliseq for Illumina custom NGS panel was designed using the Illumina Design Studio tool (Illumina, San Diego, USA) to target the hotspot exons of 22 genes known to be frequently mutated in MPNs, namely *ABL1, ASXL1, CALR, CBL, CEBPA, CSF3R, DNMT3A, EZH2, FLT3, IDH1, IDH2, JAK2, KIT, MPL, NPM1, PDGFRA, RUNX1, SF3B1, SRSF2, TET2, TP53,* and *U2AF1* (Additional file 1: Table S1 and Additional file 2: Table S2).

### Technical validation of custom NGS panel performance

The custom NGS panel performance was validated using four reference standards through two identical but independent NGS runs (Additional file 3: Table S3). The reference standards used were: (1) Tru-Q0 (100% wild-type) Reference Standard (Horizon, Cambridge, UK)—as a negative control; (2) Tru-Q1 (5% Tier) Reference Standard (Horizon)—as a positive control for 5% mutant allele in *FLT3* (p.ΔI836), *IDH1* (p.R132C), and *JAK2* (p.V617F); 3) Tru-Q7 (1.3% Tier) Reference Standard (Horizon)—as a positive control for 1.3% mutant allele in *FLT3* (p.D835Y, p.ΔI836), *IDH1* (p.R132C/H), *IDH2* (p.R140Q, p.R172K), *JAK2* (p.V617F), *KIT* (p.D816V), and *PDGFRA* (p.D842V); and 4) Seraseq™ Myeloid Mutation DNA Mix (SeraCare, Milford, USA)—as a positive control for mutant allele in *ABL1* (p.T315I), *ASXL1* (p.E635fs*15, p.G646fs*12), *CALR* (p.L367fs*46), *CBL* (p.R420Q, p.L380P), *CEBPA* (p.H24fs*84, p.K313_V314insK), *CSF3R* (p.T618I), *FLT3* (c.1759_1800dup, duplication of chr13:28,608,250–28,608,277, p.D835Y), *IDH1* (p.R132C), *JAK2* (p.V617F, p.N542_E543del), *MPL* (p.W515L), *NPM1* (p.W288fs*12), *SF3B1* (p.K700E, p.K666N), *SRSF2* (p.P95_R102del), and *U2AF1* (p.S34F) (Additional file 3: Table S3). The NGS assay performance was assessed based on the sensitivity, specificity, repeatability, concordance, and positive predictive values.

### Study cohort

Ten patients who were clinically diagnosed with ET, PV or PMF according to the 2016 WHO classification guidelines [4] in the participating institutions were recruited for this study. All patients who agreed to participate in the study provided their written consent. Approximately 10 mL peripheral blood samples from each patient were collected in EDTA tubes and processed within 24 h to extract the DNA from the cells. Patient demographic and clinical data were recorded by using standardised forms.

### Genomic DNA extraction and quality control

Genomic DNA was extracted from each blood sample using the Maxwell**®** RSC Whole Blood DNA Kit (Promega, Madison, USA) according to the manufacturer’s guidelines. The purity (A_260_/A_280_ and A_260_/A_230_) and concentration of extracted DNA were assessed using the Nanodrop™ (Thermo Fisher Scientific, Waltham, USA) spectrophotometer. DNA samples with satisfactory purity ratios of 1.80–2.00 (A_260_/A_280_) and 2.00–2.20 (A_260_/A_230_) were visualised using 1% agarose gel electrophoresis and stored for downstream experiments.

### Preparation of NGS libraries and sequencing

The NGS libraries were prepared according to the AmpliSeq for Illumina On-Demand, Custom and Community Panels Reference Guide Protocol, DNA Panels Standard Workflow Procedure for Three Primer Pools, using reagents from the AmpliSeq™ Library PLUS for Illumina®. The concentration of the input DNA was measured using the Qubit dsDNA BR Assay and the concentration of the constructed libraries was assessed using the Qubit dsDNA HS Assay on the Qubit 2.0 Fluorometer (Thermo Fisher Scientific) following the manufacturer’s protocol. The quality of the constructed libraries was assessed using the DNA Hi Sens Lab Chip (PerkinElmer, Waltham, USA) according to the DNA High Sensitivity Assay User Guide for LabChip GX Touch/GXII Touch Standard Sample Workflow. Libraries were denatured and diluted according to the MiSeq System Denature and Dilute Libraries Guide Protocol A: Standard Normalization Method for MiSeq Reagent Kit v2, with a PhiX library used as a sequencing control. Pooled sequencing of 6 samples per run was conducted on an Illumina Miseq using the MiSeq Reagent Micro Kit v2 (2 × 150 base pair (bp), paired end) to a minimum coverage depth of 5000 × according to the manufacturer’s protocol.

### Quality control and bioinformatics analysis of NGS data

**Fig. 1:**
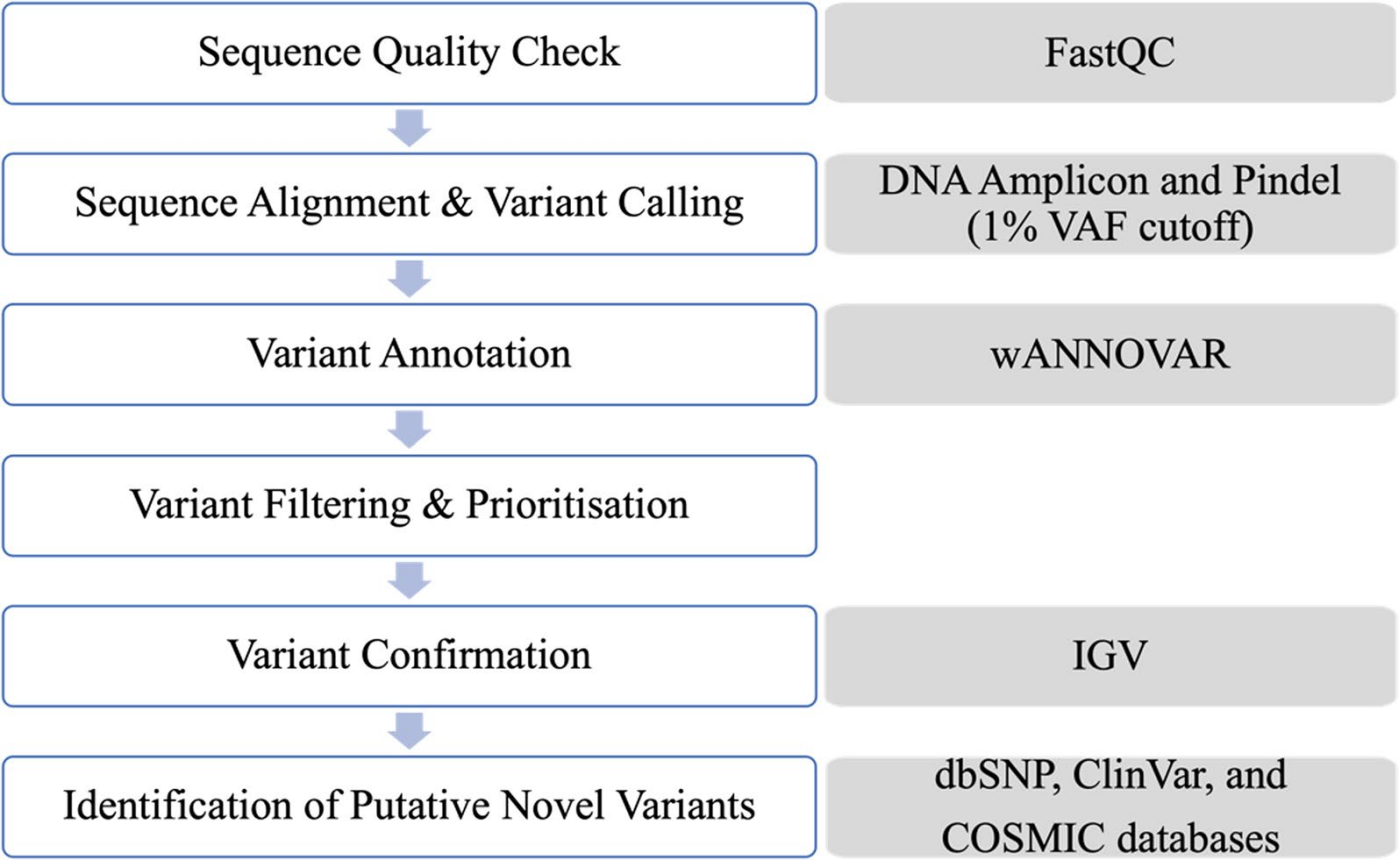
Schematic representation of steps involved in the quality control and bioinformatics analysis of the NGS data. The variant filtering and prioritisation step is further illustrated in Fig. 3. Adapted from Dai et al., 2019 [11] and Zheng et al., 2018 [12]

Figure 1 shows a schematic representation of the steps involved in the quality control and bioinformatics analysis of the NGS data. The sequencing metrics were visualized using the Illumina Sequence Analysis Viewer software. The quality of the raw NGS data was assessed using the FastQC software on the Illumina BaseSpace™ Sequence Hub. The sequencing data was analysed using a combination of two sequence alignment and variant calling applications (apps) also on the Illumina BaseSpace™ Sequence Hub—the DNA Amplicon app and Pindel app, at a 1% somatic variant allele frequency (VAF) detection limit, aligned against the Genome Reference Consortium human genome build 37 (GRCh37). The DNA Amplicon app is designed to detect small variants, whereas the Pindel app is designed to detect larger structural variants such as large deletions (as large as 10 kb), medium sized insertions, inversions and tandem duplications [10]. The DNA Amplicon app also generates a detailed report that summarises various information including sample read quality, amplicon data, base level statistics, and coverage by amplicon region. The called variants were then annotated using wANNOVAR (Wang Genomics Lab, Philadelphia, USA).

For the technical validation of the custom NGS panel, only variants that were present in the four reference standards were filtered-in and prioritized; whereas for the screening of the clinical MPN samples, all annotated variants were manually reviewed by filtering and prioritizing using the following criteria: (1) all variants except for exonic variants were excluded, (2) variants with minor allele frequencies (MAFs) of ≥ 1% (as reported in the 1000 Genomes Project, ExAC, ESP6500, and gnomAD databases) were excluded, and (3) potential sequencing errors (variants with VAF of < 5% and/or appear in majority of the samples) were excluded. In addition, variants with VAFs between 1 and 5% were inspected to check for any pathogenic/likely pathogenic variants. Aligned read (.bam) files for all samples were then manually inspected to confirm the presence of the filtered-in and prioritized variants using the Integrative Genomics Viewer (IGV) software (Broad Institute, Cambridge, USA). In order to identify any putative novel variants, the variants were manually checked against dbSNP [13] as well as the ClinVar [14] and COSMIC [15] databases to determine if they had been previously reported as pathogenic.

### Variant confirmation via Sanger sequencing

The Ensembl genome browser [16] and NCBI Primer-BLAST [17] were used to design PCR primers for the target variants (primer sequences are listed in Additional file 4: Table S4). Each PCR mixture contained 17 μL of nuclease free water, 25 μL of the Amplitaq Gold® 360 Master Mix (Thermo Fisher Scientific), 2 μL 360 GC Enhancer (Thermo Fisher Scientific), 2 μL each of 5 μM forward and reverse primer, and 2 μL of 50 ng/μL sample DNA. For the *TET2* exon 7 p.D1314Mfs*48 variant, the 360 GC Enhancer in the PCR mixture was found to reduce the PCR product yield, and was therefore replaced with nuclease free water. The PCR thermocycling conditions for all NGS detected variants were as follows: 95 °C for 10 min, 35 cycles at 95 °C for 30 s, 55 °C for 30 s, and 72 °C for 1 min, followed by a final extension at 72 °C for 7 min. The PCR products were visualised using a 1% agarose gel electrophoresis to confirm successful amplification and then purified using Wizard® SV Gel and PCR Clean-Up System (Promega) by centrifugation according to the manufacturer’s instructions but eluted in 15 mL of nuclease free water twice to obtain higher product concentration. The concentration and purity of the PCR products were assessed using the Nanodrop™ spectrophotometer as previously described. Samples with satisfactory concentration and purity ratios were sent for Sanger sequencing. Alignment of Sanger sequence (.seq) files were conducted via the ClustalW algorithm using the Jalview software [18] whereas the Sanger sequence chromatograms were analysed using the 4peaks software (Nucleobytes, Aalsmeer, Netherlands). The pathogenicity of the variants was assessed using ClinVar [14].

## Results

### Technical validation of the custom NGS panel

The custom NGS panel performance was evaluated using a set of reference standards in two identical but independent NGS runs. After library preparation, all samples were confirmed to be of sufficient quality and quantity. The majority of the constructed libraries were within the targeted size range of 400 bp for sequencing using the custom NGS panel (Additional file 5: Fig. S1). Both NGS runs achieved cluster densities of 860 to 878 K/mm^2^, > 93% of clusters passed the quality filter, and > 95% of the read bases with quality scores of above Q30, which were close to The Miseq System specifications of 865–965 K/mm^2^ cluster density and > 80% of bases above Q30 (Additional file 6: Table S5). All samples achieved ~ 99.5% on-target aligned reads and minimum amplicon mean coverage depths of between 4589x to 7944x (Additional file 7: Table S6). Analysis of the coverage depth per amplicon region revealed that 98.6% of the targeted regions (n = 216/219 amplicons) had average coverage depths of > 1000x. Two amplicons had average coverage depths of < 1000x, namely AMPL89337 (*DNMT3A* exon 17, chr2:25464411–25464625) and AMPL1156 (*TP53* exon 4/exon 5, chr17:7578360–7578579), while AMPL117202 (*MPL* exon 10, chr1:43814902–43815103) had a coverage depth of below 100x (Additional file 8: Fig. S2).

**Fig. 2:**
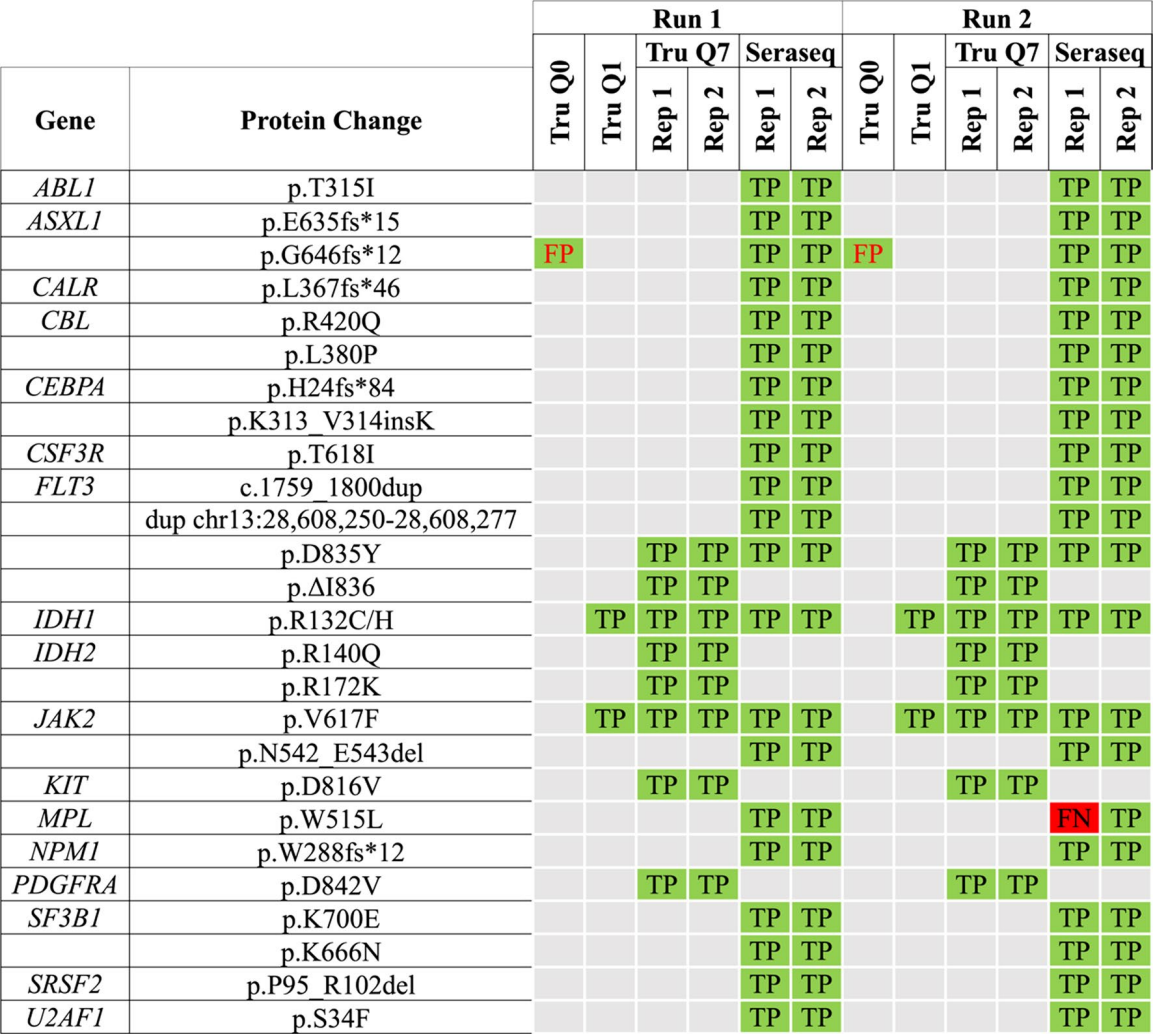
Combined analysis of variants with the DNA Amplicon and Pindel apps to evaluate the performance of the custom 22-gene NGS panel based on two identical but independent NGS runs using reference standards. The *MPL* W515L variant in the Seraseq Myeloid Mutation Mix (Seraseq) was not detected in one of the replicates, giving the custom NGS panel a sensitivity of 99.2%. One variant was detected in the wild-type reference standard TruQ0, giving the panel a specificity of 96.3%. The custom NGS panel also had a positive predictive value of 97.7%, an average intra-run and inter-run concordance of 98.8% [range 95.2–100%] and 99.0% [range 95.2–100%] respectively, and was able to detect variants at as low as 1% allele frequency. FP, False positive; TP, True positive; FN; False negative; Rep 1, Replicate 1; Rep 2, Replicate 2

Combined analysis of sequencing results with the DNA Amplicon and Pindel apps revealed that the former was able to detect all known variants in the reference standards except for large duplications in *FLT3*; whereas Pindel was able to detect all frameshift variants as well as large duplications in *FLT3*, but not SNVs (Fig. 2). Overall, the custom NGS panel has a sensitivity of 99.2%, a specificity of 96.3%, a positive predictive value of 97.7%, an average intra-run concordance of 98.8% [range 95.2–100%], an average inter-run concordance of 99.0% [range 95.2–100%], and a detection limit of 1% VAF (Fig. 2).

### Study cohort demographics and clinical data

A total of 10 MPN patients (ET n = 3, PV n = 3, PMF n = 4) were recruited for this study (Sunway Medical Centre (n = 7), Ampang Hospital (n = 1) and Universiti Kebangsaan Malaysia Medical Centre (n = 2)) (Table 1). All patients were diagnosed based on the latest WHO criteria [4]. The median age of diagnosis was 52 years (range: 30–79 years). The majority of the patients were male (n = 7/10). Half of the study cohort was of Chinese ethnicity, 4 were Malay, and 1 was Dutch. Six patients presented with constitutional symptoms, and only 1 patient (Sample 09, overt-PMF) presented with hepatosplenomegaly. Half of the study cohort had a history of smoking. Seven patients were tested positive for the *JAK2* V617F driver mutation, and 3 were positive for *CALR* driver mutations. No patients tested positive for *MPL* driver mutations.

**Table 1:** Study Sample demographics and clinical data

Sample	MPN subtype	Age (years)	Sex	Ethnicity	Hb (g/dL)	WBC (× 10^9^/L)	Platelet (× 10^9^/L)	HSM	Constitutional symptoms	Smoker	NGS-detected variants (VAF%)
01	ET	30	M	Malay	17.1	7.6	1730	No	No	No	*CALR* p.Lys385Ilefs*46 (35.2%) *CALR* p.Lys385Thr (35.5%)
											*TET2* p.Ile1195Val (49.6%)
02	ET	50	F	Chinese	13.2	10.9	776	No	No	No	*CALR* p.Lys385Asnfs*46 (15.9%) *TET2* p.Glu1513Gly (51.6%)
											*TET2* p.Phe868Leu (48.7%) *TET2* p.Tyr1631* (1.4%)^b^
03	ET	79	M	Malay	11.1	24.5	869	No	No	Yes	*JAK2* p.Val617Phe (88.0%) *ASXL1* p.Gln1433Gln (49.2%)^a^
											*DNMT3A* p.Pro385Pro (51.6%)^c^
04	PV	54	M	Chinese	18.9	17.2	426	No	Yes	Yes	*JAK2* p.Val617Phe (72.9%) *ASXL1* p.Gly646Trpfs*10 (31.3%)^a^
05	PV	66	F	Chinese	19.3	18.1	423	No	Yes	No	*JAK2* p.Val617Phe (66.5%)
06	PV	73	M	Dutch	20.0	9.0	334	No	Yes	Yes	*JAK2* p.Val617Phe (19.2%) *U2AF1* p.Gln157Pro (9.8%)
07	Pre-PMF	50	M	Chinese	15.0	17.8	859	No	Yes	Yes	*JAK2* p.Val617Phe (12.1%) *ABL1* p.Asn350Ser (48.8%)
08	Pre-PMF	50	F	Malay	8.7	58.4	1099	No	Yes	No	*JAK2* p.Val617Phe (53.7%) *RUNX1* p.Gln308His (49.6%)^c^
											*SF3B1* p.Lys700Glu (45.6%)
											*TET2* p.Asp1314Metfs*48 (46.3%)
											*TET2* p.Tyr1245Cys (44.9%)^a^
09	Overt-PMF	46	M	Malay	7.1	3.9	452	Yes	No	No	*CALR* p.Leu367Thrfs*45 (10.6%) *ASXL1* p.Leu731Tyrfs*12 (38.5%)^a^
10	Overt-PMF	63	M	Chinese	7.3	5.5	54	No	Yes	Yes	*JAK2* p.Val617Phe (12.2%) *ASXL1* p.Tyr591* (40.8%)
											*TET2* p.Ala304Val (50.1%)
											*U2AF1* p.Gln157Pro (42.5%)

Hb, haemoglobin; WBC, white blood cell; HSM, hepatosplenomegaly

^a^Putative novel variant

^b^Variant with low allele frequency, not validated via Sanger sequencing

^c^Variant of uncertain significance (VUS)/likely benign/benign variant included for validation of the custom NGS panel

### Identification of genetic variants in clinical MPN samples

**Fig. 3:**
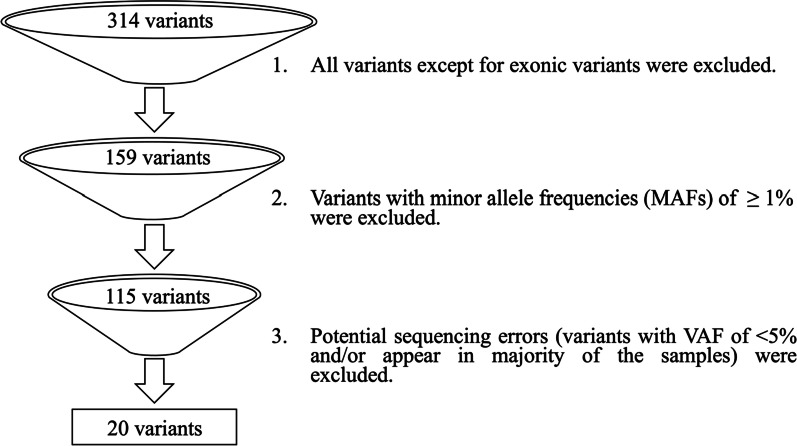
Variant filtering and prioritization process. Note that after this process, variants with VAFs between 1 and 5% were inspected for the presence of any pathogenic/likely pathogenic variants. Adapted from Zheng et al., 2018 [12]

An initial total of 314 unique variants were detected across all 10 clinical MPN samples (Fig. 3). After filtering out intronic and UTR variants as well as all variants with MAFs of ≥ 1%, 115 exonic variants remained. Subsequently, variants determined as sequencing errors were excluded. Ultimately, a total of 20 unique variants (which include known MPN driver mutations) with VAFs above 5% were identified across the 10 clinical MPN samples, and one variant with a VAF of 1.4% was found to be pathogenic as listed in the COSMIC database (COSM97191) (Table 2).

**Table 2:** Details of all NGS detected variants across the 10 clinical MPN samples

Gene	c.DNA	Aa change	Conse-quence	dbSNP	ClinVar assertion/COSMIC ID	Sample	VAF (%)	Sanger
*CALR*	c.1092_1143del	p.Leu367Thrfs*45	Fs del	NA	Pathogenic/COSM1738055	09, Overt-PMF	10.6	✓
*CALR*	c.1154_1155insTTGTC	p.Lys385Asnfs*46	Fs ins	rs765476509	COSM1738056	02, ET	15.9	✓
*CALR*	c.1153_1154insTATGT	p.Lys385Ilefs*46	Fs ins	NA	COSM5985669	01, ET	35.2	✓
*CALR*	c.1154A > C	p.Lys385Thr	nSNV	rs1024435400	NA	01, ET	35.5	✓
*JAK2*	c.1849G > T	p.Val617Phe	nSNV	rs77375493	Pathogenic/COSM12600	07, Pre-PMF	12.1	⨉
						10, Overt-PMF	12.2	⨉
						06, PV	19.2	✓
						08, Pre-PMF	53.7	✓
						05, PV	66.5	✓
						04, PV	72.9	✓
						03, ET	88.0	✓
*ABL1*	c. 1049A > G	p.Asn350Ser	nSNV	rs144448357	NA	07, Pre-PMF	48.8	✓
*ASXL1*	c.1927_1928insGGGGGGGGTG GCCCGGGTGGAGGTGGCGG CGGGGCCACCGATGAGGGG GGGGGCAGAGGCAGCAGCA	p.Gly646Trpfs*10^a^	stopgain	rs750318549	NA	04, PV	31.3	✓
*ASXL1*	c.1772dupA	p.Tyr591*	stopgain	rs762036456	COSM4169775, COSM4169776	10, Overt-PMF	40.8	✓
*ASXL1*	c.2190del	p.Leu731Tyrfs*12^a^	Fs del	NA	NA	09, Overt-PMF	38.5	✓
*ASXL1*	c.4299A > G	p.Gln1433Gln^a^	sSNV	NA	NA	03, ET	49.2	✓
*DNMT3A*	c.1155G > A	p.Pro385Pro^c^	sSNV	rs368009374	VUS/likely benign	03, ET	51.6	NA
*RUNX1*	c.924G > T	p.Gln308His^c^	nSNV	rs80314254	Benign	08, Pre-PMF	49.6	✓
*SF3B1*	c.2098A > G	p.Lys700Glu	nSNV	rs559063155	Likely pathogenic/ COSM84677	08, Pre-PMF	45.6	✓
*TET2*	c.911C > T	p.Ala304Val	nSNV	NA	COSM5610834, COSM5610835	10, Overt-PMF	50.1	✓
*TET2*	c.2604T > G	p.Phe868Leu	nSNV	rs147836249	COSM87107	02, ET	48.7	✓
*TET2*	c.3583A > G	p.Ile1195Val	nSNV	rs568009712	NA	01, ET	49.6	✓
*TET2*	c.3734A > G	p.Tyr1245Cys^a^	nSNV	NA	NA	08, Pre-PMF	44.9	✓
*TET2*	c.3937del	p.Asp1314Metfs*48	Fs del	NA	COSM4383928	08, Pre-PMF	46.3	✓
*TET2*	c.4893T > A	p.Tyr1631*^b^	stopgain	NA	COSM97191	02, ET	1.4	NA
*TET2*	c.4538A > G	p.Glu1513Gly	nSNV	rs553669299	NA	02, ET	51.6	✓
*U2AF1*	c.470A > C	p.Gln157Pro	nSNV	rs371246226	Likely pathogenic/	06, PV	9.8	⨉
				rs371246226	COSM211534, COSM1318797	10, Overt-PMF	42.5	✓

Aa change, amino acid change; Fs del, frameshift deletion; Fs ins, frameshift insertion; ✓, Sanger detected; ⨉, Sanger undetected; NA, data not available

^a^Putative novel variant

^b^Variant with low allele frequency, not validated via Sanger sequencing

^c^Variant of uncertain significance (VUS)/likely benign/benign variant included for validation of the custom NGS panel

**Fig. 4:**
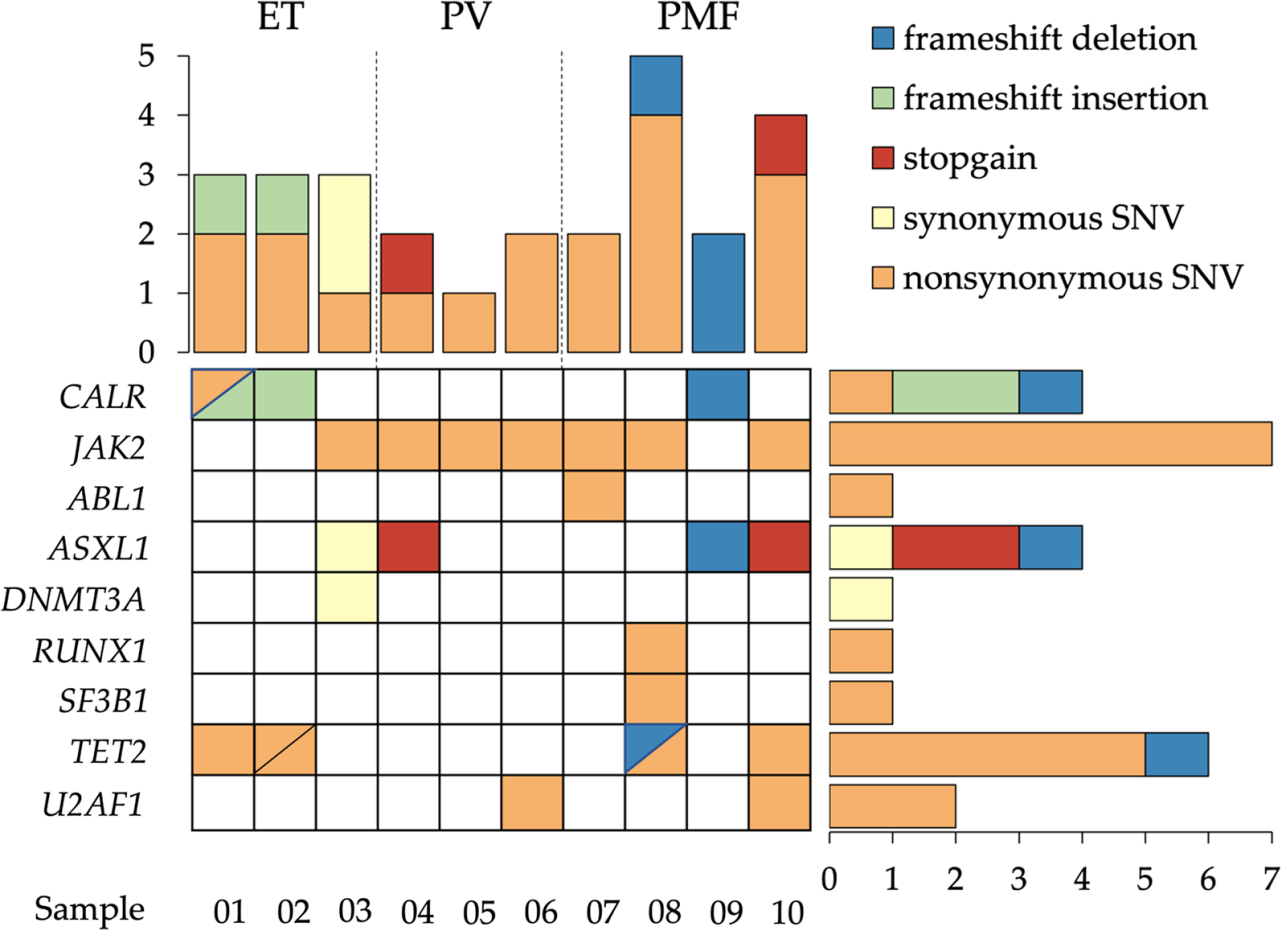
Variants detected in the clinical MPN samples with VAF > 5%. Note that Sample 02 also carries another variant in *TET2* with a VAF of 1.4% (not shown in Fig. 4)

On average, the PMF samples appeared to harbour the highest number of variants, whereas the PV samples appeared to harbour the least number of variants (Fig. 4). Among the 20 unique variants with VAF > 5%, 13 were SNVs (synonymous SNV (sSNV) n = 2, nonsynonymous SNV (nSNV) n = 11) and 7 were indels (frameshift insertion (fs ins), n = 2; frameshift deletions (fs del), n = 3; stopgain, n = 2). Out of the 10 sequenced clinical samples, the *JAK2* V617F driver mutation was identified in 7 samples, while *CALR* driver mutation was identified in 3 samples. Aside from driver mutations, other variants were also detected, including an nSNV in *CALR* as well as variants in *ABL1* (n = 1), *ASXL1* (n = 4), *DNMT3A* (n = 1); *RUNX1* (n = 1), *SF3B1* (n = 1), *TET2* (n = 6), and *U2AF1* (n = 2) (Fig. 4). All NGS-detected variants with allele frequencies of ≥ 15% were confirmed via Sanger sequencing (Table 2), except for the *DNMT3A* sSNV that was not confirmed due to nonspecific amplification (Additional file 9: Fig. S3).

The *ABL1* p.Asn350Ser point mutation identified in this study (Sample 07, pre-PMF) was reported in dbSNP (rs144448357). However, it is unknown whether it was previously identified in MPN due to the lack of ClinVar and COSMIC data (Table 2). The variant was identified in a patient diagnosed with pre-PMF at 50 years of age, with *JAK2* V617F mutation. The patient presented with increased platelet (859 × 10^9^/L) and white cell counts (17.8 × 10^9^/L), as well as constitutional symptoms (Table 1).

Four *ASXL1* variants were identified in this study, of which, the *ASXL1* p.Gly646Trpfs*10 (c.1927_1928insGGGGGGGGTGGCCCGGGTGGAGGTGGCGGCGGGGCCACCGATGAGGGGGGGGGCAGAGGCAGCAGCA) stopgain variant (Sample 04, PV) was found to be located in the same dbSNP cluster rs750318549 as the previously reported *ASXL1* p.Gly646Trpfs*12 *(*c.1934dupG) stopgain, which is the most common *ASXL1* mutation accounting for > 50% of all identified *ASXL1* mutations in myeloid malignancies [19] (Table 2). While *ASXL1* p.Gly646Trpfs*12 is the result of a duplication of a G nucleotide within a homopolymer region of eight G nucleotides [19], the variant *ASXL1* p.Gly646Trpfs*10 identified in this study is the result of an insertion of 67 nucleotides at position chr20: 31022442, making it a novel stopgain variant. Two other *ASXL1* variants identified in this study, *ASXL1* p.Leu731Tyrfs*12 (Sample 09, overt-PMF) and Q1433Q (Sample 03, ET) were also putative novel variants with no dbSNP, COSMIC or ClinVar data; whereas the *ASXL1* p.Tyr591*variant (Sample 10, overt-PMF) has been reported in various diseases including ET and MF [9, 20], MDS [21], chronic myelomonocytic leukaemia [22], AML [23], mast cell neoplasm [24] and CNL [25], as well as breast cancer [26], but has not been previously reported in PV (Table 2).

Seven *TET2* variants were identified in this study, of which, *TET2* p.Tyr1245Cys (Sample 08, pre-PMF) was not found to be reported in dbSNP, COSMIC or the ClinVar database. The *TET2* p.Ala304Val (Sample 10, overt-PMF) and p.Phe868Leu (Sample 02, ET) variants have not been previously reported in MPN. *TET2* p.Ala304Val has only been previously identified in melanoma [27], while *TET2* p.Phe868Leu has been previously identified in estrogen- and progesterone-receptor positive breast cancer [28], adult T cell lymphoma/leukaemia [29], and MDS [30, 31]. The *TET2* p.Asp1314Metfs*48 variant (Sample 08, pre-PMF) has been reported in ET [32] and MDS [21]. Two other variants were reported in dbSNP—*TET2* p.Ile1195Val (rs568009712) (Sample 01, ET) and p.Glu1513Gly (rs553669299) (Sample 02, ET), but the associated disease(s) are unknown. Of note, Sample 02 was found to carry another *TET2* variant—the p.Tyr1631* stopgain, reported as pathogenic in the COSMIC database and previously identified in angioimmunoblastic T cell lymphoma [33], CML, and AML [34] at a VAF of 1.4% (Table 2).

Two of the most common mutations in *SF3B1* and *U2AF1* were identified in this study, namely *U2AF1* p.Gln157Pro (rs371246226, COSM211534, COSM1318797) and *SF3B1* p.Lys700Glu (rs559063155, COSM84677) [35–37]. Both variants have been reported to be likely pathogenic in the ClinVar database. *SF3B1* p.Lys700Glu was found in a pre-PMF sample (Sample 08) which also harboured the *TET2* p.Tyr1245Cys and *TET2* p.Asp1314Metfs*48 variants alongside the *JAK2* V617F driver mutation. The patient was 50 years of age and presented with abnormally high platelet count (1099 × 10^9^/L), anaemia (Hb = 8.7 g/dL), and constitutional symptoms (Table 1). *U2AF1* p.Gln157Pro was identified in PV and overt-PMF (Sample 06 and Sample 10, respectively) (Table 2). The overt-PMF sample (Sample 10) also harboured the *ASXL1* p.Tyr591* stopgain variant in addition to the *JAK2* V617F driver mutation. The patient was 63 years of age and presented with severe anaemia (Hb = 7.3 g/dL), leucopenia (WBC = 5.5 × 10^9^/L) and thrombocytopenia (Platelet = 54 × 10^9^/L), with constitutional symptoms (Table 1).

## Discussion

In this study, we evaluated the performance of our custom 22-gene NGS panel using reference standards and subsequently, a small cohort of clinical MPN samples. The 22 genes selected in this panel are known to be frequently mutated in myeloid neoplasms (not necessarily MPNs), and are known disease markers with diagnostic, prognostic and/or therapeutic value. First, the performance of the custom NGS panel was technically validated using reference standards in two identical but independent sequencing runs. The combined analysis of variants detected by the DNA Amplicon and Pindel tools revealed that the panel achieved high sensitivity, specificity, concordance and positive predictive values. The overall good performance of the panel was further supported in its ability to detect variants with VAF values as low as 1% (Fig. 2). Overall, the depth-of-coverage achieved across all amplicons was around our targeted coverage of 5000x (Additional file 8: Fig. S2). However, the low average depth-of-coverage of the *MPL* amplicon (AMPL117202, *MPL* exon 10, chr1:43814902–43815103) at < 100 × could potentially lead to false negative results. In order for the panel to be used in MPN diagnostics, optimisation of the sequencing coverage for the *MPL* region is critical for the detection of driver mutations in *MPL*. In addition, AMPL89337 (*DNMT3A* exon 17, chr2:25464411–25464625), AMPL1156 (*TP53* exon 4/exon 5 chr17:7578360–7578579) and AMPL90417 (*ASXL1* exon 13, chr20:31022935–31023187) were found to have average depths-of-coverage of < 1000x (Additional file 8: Fig. S2, Additional file 10).

Uneven coverage or coverage bias may originate from various steps in the NGS workflow. The use of the PCR method during NGS library preparation has been found to be the primary contributor [38–40]. In PCR, the annealing efficiency of primers is commonly hindered by templates that are GC-rich that tend to remain double-stranded, as well as templates that are AT-rich (or GC-poor) that anneal poorly to primers. As a result, GC-and AT-rich regions (also known as low-complexity regions) are often poorly amplified and manifest in NGS as regions with low sequencing coverage. Further analysis of AMPL117202 showed that it had a GC content of 61% and AT content of 39%, and a plot of the nucleotide distribution revealed that the GC content was > 60% in the majority of 30 bp windows (Additional file 11: Fig. S4). Hence, it is likely that the majority of the AMPL117202 amplicons remained annealed as double-stranded DNA during PCR, leading to inefficient amplification and subsequent low NGS coverage of the amplicons. Future studies should involve the review of targeted regions and further optimisation of the NGS library preparation to improve the coverage of GC-rich regions, i.e. optimising PCR amplification conditions by using lower primer-extension temperature [38]. In order to rule out false negatives due to coverage bias, variants located in *DNMT3A* exon 17 and *TP53* exon 4/exon 5 should also be used to validate the performance of the custom panel.

From the screening of clinical MPN samples with the custom NGS panel, 21 unique variants including MPN driver mutations in *JAK2* and *CALR*, and one pathogenic stopgain variant in *TET2* with a VAF of 1.4% were identified. The *ASXL1* p.Leu731Tyrfs*12, p.Gln1433Gln, p.Gly646Trpfs*10 and *TET2* p.Tyr1245Cys variants were identified as putative novel variants, whereas reported and likely pathogenic variants were identified in *ABL1*, *SF3B1* and *U2AF1*. The study findings support the notion that the co-presence of multiple variants within a single sample results in a potentially synergistic effect that promotes disease development and progression, and contributes towards higher symptom burden, poorer prognosis, higher risk of leukaemic transformation, and drug resistance [21, 41–46]. In *ABL1*, point mutations that confer resistance against tyrosine kinase inhibitors in patients with CML have been discovered in more than 50 different hotspots in the kinase domain [47–50]. It is possible that *ABL1* p.Asn350Ser identified in this study may confer drug resistance in a similar fashion to those that have previously been identified.

As MPNs are a clonally heterogenous and multifactorial group of diseases, differences in gene dosage and clonal architecture, the order of mutation acquisition and even germline predisposition can contribute towards MPN pathogenesis as well as differences in disease phenotype and prognosis [51, 52]. In addition, other factors such as lineage bias in haematopoietic stem cells, changes in the bone marrow microenvironment, and aging have also been reported [53]. Further studies should aim to experimentally characterise the functional impact of the variants identified and investigate their possible synergistic effects and biological significance in myeloid disorders, especially in MPN. The putative novel variants should be further investigated using in vitro candidate gene approaches via protein function assays and bioinformatics analyses in order to elucidate their impact especially in poorly characterized proteins [54, 55].

In this study, all NGS-identified variants were confirmed via bidirectional Sanger sequencing except for *TET2* p.Tyr1631*, *JAK2* V617F and *U2AF1* p.Gln157Pro where the allelic frequencies were < 15%, whereas *DMNT3A* p.Pro385Pro was not confirmed due to nonspecific primer binding during the PCR step (Table 2, Additional file 9: Fig. S3). Sanger sequencing is widely regarded as the ‘gold standard’ for the validation of NGS-detected variants as it can discriminate true variants from NGS artifacts or sequencing errors [56]. However, as the Sanger method relies on the detection of fluorescence, it has limited sensitivity when detecting variants with low allele frequencies; particularly in mosaic tumour samples, leading to false negative results [57–59]. The sensitivity of Sanger sequencing has been reported to be around 15–20% allele frequency, whereas NGS has a sensitivity of approximately 1% allele frequency [59, 60]. Therefore, best practice standards for NGS variant confirmation should include alternative methods with higher sensitivity, such as droplet digital PCR or allele-specific qPCR, followed by high-resolution melting curve analysis for the confirmation of variants with low allele frequencies [56, 60, 61]. Nevertheless, such methods are also accompanied by technical limitations and caveats which should be addressed and optimised for their specific intended purposes [62].

Future studies may also benefit from the use of an expanded bioinformatics pipeline which will ultimately provide a greater wealth of information to the clinician, such as the indication of variant germline/somatic status, the identification of clonal haematopoiesis of indeterminate potential (CHIP, which shows evidence of clonal expansion), monitoring of minimal residual disease, determination of disease predisposition, as well as accurate prediction of treatment response/outcome [63, 64]. A larger study cohort as well as the collection of samples and clinical data at follow-up as well as data on response to therapy and survival will allow for better genotype–phenotype associations and contribute towards the better understanding of MPNs.

## Conclusions

In summary, the custom NGS panel enabled the detection of known MPN-associated genetic variants, as well as the identification of novel variants of potential biological significance, indicating its potential clinical utility in the genetic profiling of MPN patients. However, further performance optimisation is required especially for regions with poor coverage depth to ensure that the custom NGS panel will serve as a robust and reliable tool for the personalised management of MPNs. It is hoped that the data generated from the screening of MPN patients using the custom NGS panel will also contribute towards the MPN knowledgebase, and support the adoption of more accurate genomics-based disease classification as well as prognostic frameworks for the management of MPNs.

## Supplementary Information

**Additional file 1.** **Table S1.** Targeted exons of 22 MPN-associated genes in the custom NGS panel.

**Additional file 2.** **Table S2.** Amplicon regions.

**Additional file 3.** **Table S3.** Details of variants present in reference standards.

**Additional file 4.** **Table S4.** Primers used in variant confirmation.

**Additional file 5.** **Fig. S1.** Representative image of (**a**) Labchip gel and (**b**) multiple overlay electropherogram of the constructed libraries of the reference standards. The image shows the quality and quantity of input DNA for the second NGS run for the technical validation of the custom NGS panel. The majority of the constructed libraries were within the targeted size range of 400 bp.

**Additional file 6.** **Table S5.** Sequencing metrics of NGS runs for the technical validation of the custom NGS panel.

**Additional file 7.** **Table S6.** Amplicon coverage data for the technical validation of the custom NGS panel.

**Additional file 8.** **Fig. S2.** Amplicon coverage across reference standards in the technical validation of the custom NGS panel. More details on the amplicon regions can be found in Additional file 2: Table S2 and Additional file 10.

**Additional file 9.** **Fig. S3.** Gel electrophoresis images.

**Additional file 10.** Amplicon coverage. Sheet 1 shows the genomic sequences covered by each amplicon, whereas Sheet 2 shows the depth-of-coverage per amplicon for each of the samples across the two NGS runs.

**Additional file 11.** **Fig. S4.** Distribution of GC content across AMPL117202 (*MPL* exon 10, chr1:43814902-43815103).

## Data Availability

The datasets generated and/or analysed during the current study are available in the NCBI BioProject repository, PRJNA754686.

## Abbreviations

AML: Acute myeloid leukaemia
bp: Base pair
CHIP: Clonal haematopoiesis of indeterminate potential
CML: Chronic myeloid leukaemia
CNL: Chronic neutrophilic leukaemia
COSMIC: Catalogue of somatic mutations in cancer
DNA: Deoxyribonucleic acid
dNTP: Dideoxynucleotide phosphates
ET: Essential thrombocythaemia
ExAC: Exome Aggregation Consortium
ESP6500: NHLBI Exome Sequencing Project
gnomAD: Genome Aggregation Database
IGV: Integrative genomics viewer
MDS: Myelodysplastic syndrome
MDS/MPN: Myelodysplastic/myeloproliferative neoplasms
MF: Myelofibrosis
MAF: Minor allele frequency
MPN: Myeloproliferative neoplasms
NGS: Next-generation sequencing
nSNV: Nonsynonymous single nucleotide variant
Ph: Philadelphia
PMF: Primary myelofibrosis
PV: Polycythaemia vera
SNV: Single nucleotide variant
sSNV: Synonymous single nucleotide variant
TN: Triple-negative
VAF: Variant allele frequency
