# Supplementary material for: Analysis of genetic variants in myeloproliferative neoplasms using a 22-gene next-generation sequencing panel
Source: BMC Med Genomics. 2022 Jan 15;15:10. doi: 10.1186/s12920-021-01145-0 (PMC8760696; doi:10.1186/s12920-021-01145-0)
Supplement: Supplementary file 1 — Additional file 1. Table S1. Targeted exons of 22 MPN-associated genes in the custom NGS panel. [file 12920_2021_1145_MOESM1_ESM.pdf]

Additional file 1: Table S1. Targeted exons of 22 MPN-associated genes in the custom NGS panel.

**Target Region**	**hg19 Genomic Location**
ABL1_Exon 4 (ENST00000372348);<br>ABL1_Exon 4 (ENST00000318560)	chr9:133738150-133738422
ABL1_Exon 5 (ENST00000372348);<br>ABL1_Exon 5 (ENST00000318560)	chr9:133747516-133747600
ABL1_Exon 6 (ENST00000372348);<br>ABL1_Exon 6 (ENST00000318560)	chr9:133748247-133748424
ABL1_Exon 7 (ENST00000372348);<br>ABL1_Exon 7 (ENST00000318560)	chr9:133750255-133750439
ABL1_Exon 8 (ENST00000372348);<br>ABL1_Exon 8 (ENST00000318560)	chr9:133753802-133753954
ABL1_Exon 9 (ENST00000372348);<br>ABL1_Exon 9 (ENST00000318560)	chr9:133755455-133755544
ASXL1_Exon 10 (ENST00000375687)	chr20:31019386-31019482
ASXL1_Exon 11(ENST00000375687)	chr20:31020683-31020788
ASXL1_Exon 12 (ENST00000375687)	chr20:31021087-31021720
ASXL1_Exon 13 (ENST00000375687)	chr20:31022235-31025141
CALR_Exon 9 (ENST00000316448)	chr19:13054527-13054727
CBL_Exon 8 (ENST00000264033)	chr11:119148876-119149007
CBL_Exon 9 (ENST00000264033)	chr11:119149220-119149423
CEBPA_Exon 1 (ENST00000498907)	chr19:33792244-33793320
CSF3R_Exon 10 (ENST00000373103);<br>CSF3R_Exon 10 (ENST00000373104);<br>CSF3R_Exon 10 (ENST00000373106)	chr1:36937034-36937247
CSF3R_Exon 14 (ENST00000373103);<br>CSF3R_Exon 14 (ENST00000373104);<br>CSF3R_Exon 14 (ENST00000373106)	chr1:36933423-36933563
CSF3R_Exon 15 (ENST00000373103);<br>CSF3R_Exon 15 (ENST00000373104);<br>CSF3R_Exon 15 (ENST00000373106)	chr1:36933159-36933252
CSF3R_Exon 16 (ENST00000373103);<br>CSF3R_Exon 16 (ENST00000373104);<br>CSF3R_Exon 16 (ENST00000373106)	chr1:36932831-36932912
CSF3R_Exon 17 (ENST00000373103);<br>CSF3R_Exon 17 (ENST00000373106)	chr1:36931958-36932509
CSF3R_Exon 18 (ENST00000373104)	chr1:36931697-36931801
DNMT3A_Exon 1 (ENST00000380746)	chr2:25475063-25475066
DNMT3A_Exon 2 (ENST00000380746)	chr2:25472526-25472593
DNMT3A_Exon 2 (ENST00000321117);<br>DNMT3A_Exon 2 (ENST00000264709);<br>DNMT3A_Exon 2 (ENST00000380746)	chr2:25536782-25536853
DNMT3A_Exon 3 (ENST00000321117);<br>DNMT3A_Exon 3 (ENST00000264709);<br>DNMT3A_Exon 3 (ENST00000380746)	chr2:25523008-25523112

Additional file 1: Table S1. *Cont.*

DNMT3A_Exon 4 (ENST00000321117); DNMT3A_Exon 4 (ENST00000264709); DNMT3A_Exon 4 (ENST00000380746)	chr2:25505257-25505580
DNMT3A_Exon 5 (ENST00000321117); DNMT3A_Exon 5 (ENST00000264709)	chr2:25498369-25498412
DNMT3A_Exon 6 (ENST00000321117); DNMT3A_Exon 6 (ENST00000264709)	chr2:25497810-25497956
DNMT3A_Exon 7 (ENST00000321117); DNMT3A_Exon 7 (ENST00000264709); DNMT3A_Exon 3 (ENST00000380746)	chr2:25470906-25471121
DNMT3A_Exon 8 (ENST00000321117); DNMT3A_Exon 8 (ENST00000264709); DNMT3A_Exon 4 (ENST00000380746)	cchr2:25470460-25470618
DNMT3A_Exon 9 (ENST00000321117); DNMT3A_Exon 9 (ENST00000264709); DNMT3A_Exon 5 (ENST00000380746)	chr2:25469920-25470027
DNMT3A_Exon 10 (ENST00000321117); DNMT3A_Exon 10 (ENST00000264709); DNMT3A_Exon 6 (ENST00000380746)	chr2:25469489-25469645
DNMT3A_Exon 11 (ENST00000321117); DNMT3A_Exon 11 (ENST00000264709); DNMT3A_Exon 7 (ENST00000380746)	chr2:25469029-25469178
DNMT3A_Exon 12 (ENST00000321117); DNMT3A_Exon 12 (ENST00000264709); DNMT3A_Exon 8 (ENST00000380746)	chr2:25468889-25468933
DNMT3A_Exon 13 (ENST00000321117); DNMT3A_Exon 13 (ENST00000264709); DNMT3A_Exon 9 (ENST00000380746)	chr2:25468122-25468201
DNMT3A_Exon 14 (ENST00000321117); DNMT3A_Exon 14 (ENST00000264709); DNMT3A_Exon 10 (ENST00000380746)	chr2:25467409-25467521
DNMT3A_Exon 15 (ENST00000321117); DNMT3A_Exon 15 (ENST00000264709); DNMT3A_Exon 11 (ENST00000380746)	chr2:25467024-25467207
DNMT3A_Exon 16 (ENST00000321117); DNMT3A_Exon 16 (ENST00000264709); DNMT3A_Exon 12 (ENST00000380746)	chr2:25466767-25466851
DNMT3A_Exon 17 (ENST00000321117); DNMT3A_Exon 17 (ENST00000264709); DNMT3A_Exon 13 (ENST00000380746)	chr2:25464431-25464576
DNMT3A_Exon 18 (ENST00000321117); DNMT3A_Exon 18 (ENST00000264709); DNMT3A_Exon 14 (ENST00000380746)	chr2:25463509-25463599
DNMT3A_Exon 19 (ENST00000321117 ; DNMT3A_Exon 19 (ENST00000264709); DNMT3A_Exon 15 (ENST00000380746)	chr2:25463171-25463319

Additional file 1: Table S1. *Cont.*

DNMT3A_Exon 20 (ENST00000321117);<br>DNMT3A_Exon 20 (ENST00000264709);<br>DNMT3A_Exon 16 (ENST00000380746)	chr2:25461999-25462084
DNMT3A_Exon 21 (ENST00000321117);<br>DNMT3A_Exon 21 (ENST00000264709);<br>DNMT3A_Exon 17 (ENST00000380746)	chr2:25459805-25459874
DNMT3A_Exon 22 (ENST00000321117);<br>DNMT3A_Exon 22 (ENST00000264709);<br>DNMT3A_Exon 18 (ENST00000380746)	chr2:25458576-25458694
DNMT3A_Exon 23 (ENST00000321117);<br>DNMT3A_Exon 23 (ENST00000264709);<br>DNMT3A_Exon 19 (ENST00000380746)	chr2:25457148-25457289
EZH2_Exon 2 (ENST00000320356);<br>EZH2_Exon 2 (ENST00000350995);<br>EZH2_Exon 2 (ENST00000476773)	chr7:148544274-148544390
EZH2_Exon 3 (ENST00000320356);<br>EZH2_Exon 3 (ENST00000350995);<br>EZH2_Exon 3 (ENST00000476773)	chr7:148543562-148543690
EZH2_Exon 4 (ENST00000320356);<br>EZH2_Exon 4 (ENST00000476773)	chr7:148529726-148529842
EZH2_Exon 5 (ENST00000320356);<br>EZH2_Exon 4 (ENST00000350995);<br>EZH2_Exon 5 (ENST00000476773)	chr7:148526820-148526940
EZH2_Exon 6 (ENST00000320356);<br>EZH2_Exon 5 (ENST00000350995);<br>EZH2_Exon 6 (ENST00000476773)	chr7:148525832-148525972
EZH2_Exon 7 (ENST00000320356);<br>EZH2_Exon 6 (ENST00000350995);<br>EZH2_Exon 7 (ENST00000476773)	chr7:148524256-148524358
EZH2_Exon 8 (ENST00000320356);<br>EZH2_Exon 7 (ENST00000350995);<br>EZH2_Exon 8 (ENST00000476773)	chr7:148523546-148523724
EZH2_Exon 9 (ENST00000320356);<br>EZH2_Exon 8 (ENST00000350995);<br>EZH2_Exon 9 (ENST00000476773)	chr7:148516688-148516779
EZH2_Exon 10 (ENST00000320356);<br>EZH2_Exon 9 (ENST00000350995);<br>EZH2_Exon 10 (ENST00000476773)	chr7:148514969-148515209
EZH2_Exon 11 (ENST00000320356);<br>EZH2_Exon 10 (ENST00000350995);<br>EZH2_Exon 11 (ENST00000476773)	chr7:148514314-148514483
EZH2_Exon 12 (ENST00000320356);<br>EZH2_Exon 11 (ENST00000350995);<br>EZH2_Exon 12 (ENST00000476773)	chr7:148513776-148513870
EZH2_Exon 13 (ENST00000320356);<br>EZH2_Exon 12 (ENST00000350995);<br>EZH2_Exon 13 (ENST00000476773)	chr7:148512598-148512638

Additional file 1: Table S1. *Cont.*

EZH2_Exon 14 (ENST00000320356);<br>EZH2_Exon 13 (ENST00000350995)	chr7:148512006-148512131
EZH2_Exon 15 (ENST00000320356);<br>EZH2_Exon 14 (ENST00000350995);<br>EZH2_Exon 14 (ENST00000476773)	chr7:148511051-148511229
EZH2_Exon 16 (ENST00000320356);<br>EZH2_Exon 15 (ENST00000350995);<br>EZH2_Exon 15 (ENST00000476773)	chr7:148508717-148508812
EZH2_Exon 17 (ENST00000320356);<br>EZH2_Exon 16 (ENST00000350995);<br>EZH2_Exon 16 (ENST00000476773)	chr7:148507425-148507506
EZH2_Exon 18 (ENST00000320356);<br>EZH2_Exon 17 (ENST00000350995);<br>EZH2_Exon 17 (ENST00000476773)	chr7:148506402-148506482
EZH2_Exon 19 (ENST00000320356);<br>EZH2_Exon 18 (ENST00000350995);<br>EZH2_Exon 18 (ENST00000476773)	chr7:148506163-148506247
EZH2_Exon 20 (ENST00000320356);<br>EZH2_Exon 19 (ENST00000350995);<br>EZH2_Exon 19 (ENST00000476773)	chr7:148504738-148504798
FLT3_Exon 13 (ENST00000241453)	chr13:28608438-28608544
FLT3_Exon 14 (ENST00000241453)	chr13:28608219-28608351
FLT3_Exon 15 (ENST00000241453)	chr13:28608024-28608128
FLT3_Exon 20 (ENST00000241453)	chr13:28592604-28592726
IDH1_Exon 4 (ENST00000345146)	chr2:209113093-209113384
IDH2_Exon 4 (ENST00000330062)	chr15:90631819-90631979
JAK2_Exon 12 (ENST00000381652)	chr9:5069925-5070052
JAK2_Exon 13 (ENST00000381652)	chr9:5072492-5072626
JAK2_Exon 14 (ENST00000381652)	chr9:5073698-5073785
JAK2_Exon 15 (ENST00000381652)	chr9:5077453-5077580
JAK2_Exon 16 (ENST00000381652)	chr9:5078306-5078444
KIT_Exon 2 (ENST00000288135)	chr4:55561678-55561947
KIT_Exon 8 (ENST00000288135)	chr4:55589750-55589864
KIT_Exon 9 (ENST00000288135)	chr4:55592023-55592216
KIT_Exon 10 (ENST00000288135)	chr4:55593384-55593490
KIT_Exon 11 (ENST00000288135)	chr4:55593582-55593708
KIT_Exon 13 (ENST00000288135)	chr4:55594177-55594287
KIT_Exon 17 (ENST00000288135)	chr4:55599236-55599358
KIT_Exon 18 (ENST00000288135)	chr4:55602664-55602775
MPL_Exon 10 (ENST00000372470)	chr1:43814934-43815030
NPM1_Exon 11 (ENST00000296930);<br>NPM1_Exon 10 (ENST00000351986)	chr5:170837531-170837569
PDGFRA_Exon 12 (ENST00000257290)	chr4:55141008-55141140
PDGFRA_Exon 14 (ENST00000257290)	chr4:55144063-55144173

Additional file 1: Table S1. *Cont.*

PDGFRA_Exon 18 (ENST00000257290)	chr4:55152008-55152130
RUNX1_Exon 1 (ENST00000344691);<br>RUNX1_Exon 1 (ENST00000358356)	chr21:36259140-36259409
RUNX1_Exon 2 (ENST00000437180)	chr21:36421139-36421196
RUNX1_Exon 3 (ENST00000437180)	chr21:36265222-36265260
RUNX1_Exon 5 (ENST00000437180);<br>RUN X1_Exon 2 (ENST00000344691);<br>RUNX1_Exon 2 (ENST00000358356)	chr21:36252854-36253010
RUNX1_Exon 5 (ENST00000358356)	chr21:36193965-36193993
RUNX1_Exon 6 (ENST00000437180);<br>RUNX1_Exon 3 (ENST00000344691);<br>RUNX1_Exon 3 (ENST00000358356)	chr21:36231771-36231875
RUNX1_Exon 7 (ENST00000437180);<br>RUNX1_Exon 4 (ENST00000344691);<br>RUNX1_Exon 4 (ENST00000358356)	chr21:36206707-36206898
RUNX1_Exon 8 (ENST00000437180);<br>RUNX1_Exon 5 (ENST00000344691)	chr21:36171598-36171759
RUNX1_Exon 9 (ENST00000437180);<br>RUNX1_Exon 6 (ENST00000344691)	chr21:36164432-36164907
SF3B1_Exon 13 (ENST00000335508)	chr2:198267673-198267759
SF3B1_Exon 14 ENST00000335508)	chr2:198267280-198267550
SF3B1_Exon 15 ENST00000335508)	chr2:198266709-198266854
SF3B1_Exon 16 ENST00000335508)	chr2:198266466-198266612
SRSF2_Exon 1 (ENST00000392485)	chr17:74732881-74733242
TET2_Exon 3 (ENST00000380013);<br>TET2_Exon 3 (ENST00000305737)	chr4:106155100-106158597
TET2_Exon 4 (ENST00000380013)	chr4:106162496-106162586
TET2_Exon 5 (ENST00000380013)	chr4:106163991-106164084
TET2_Exon 6 (ENST00000380013)	chr4:106164727-106164935
TET2_Exon 7 (ENST00000380013)	chr4:106180776-106180926
TET2_Exon 8 (ENST00000380013)	chr4:106182916-106183005
TET2_Exon 9 (ENST00000380013)	chr4:106190767-106190904
TET2_Exon 10 (ENST00000380013)	chr4:106193721-106194075
TET2_Exon 11 (ENST00000380013)	chr4:106196205-106197676
U2AF1_Exon 2 (ENST00000380276)	chr21:44524425-44524512
U2AF1_Exon 6 (ENST00000380276	chr21:44514765-44514898
TP53_Exon 2 (ENST00000269305);<br>TP53_Exon 1 (ENST00000617185);<br>TP53_Exon 2 (ENST0000045526)	chr17:7579839-7579912
TP53_Exon 3 (ENST00000269305);<br>TP53_Exon 2 (ENST00000617185);<br>TP53_Exon 3 (ENST0000045526)	chr17:7579700-7579721
TP53_Exon 4 (ENST00000269305);<br>TP53_Exon 3 (ENST00000617185);<br>TP53_Exon 4 (ENST0000045526)	chr17:7579312-7579590

Additional file 1: Table S1. *Cont.*

TP53_Exon 5 (ENST00000269305);<br>TP53_Exon 5 (ENST00000622645);<br>TP53_Exon 4 (ENST00000617185);<br>TP53_Exon 4 (ENST00000610292);<br>TP53_Exon 5 (ENST00000455263)	chr17:7578371-7578554
TP53_Exon 6 (ENST00000269305);<br>TP53_Exon 6 (ENST00000622645);<br>TP53_Exon 5 (ENST00000617185);<br>TP53_Exon 5 (ENST00000610292);<br>TP53_Exon 6 (ENST00000455263);<br>TP53_Exon 2 (ENST00000510385);<br>TP53_Exon 2 (ENST00000610623);<br>TP53_Exon 2 (ENST00000619186)	chr17:7578177-7578289
TP53_Exon 7 (ENST00000269305);<br>TP53_Exon 7 (ENST00000622645);<br>TP53_Exon 6 (ENST00000617185);<br>TP53_Exon 6 (ENST00000610292);<br>TP53_Exon 7 (ENST00000455263);<br>TP53_Exon 3 (ENST00000510385);<br>TP53_Exon 3 (ENST00000610623);<br>TP53_Exon 3 (ENST00000619186)	chr17:7577499-7577608
TP53_Exon 8 (ENST00000269305);<br>TP53_Exon 8 (ENST00000622645);<br>TP53_Exon 7 (ENST00000617185);<br>TP53_Exon 7 (ENST00000610292);<br>TP53_Exon 8 (ENST00000455263);<br>TP53_Exon 4 (ENST00000510385);<br>TP53_Exon 4 (ENST00000610623);<br>TP53_Exon 4 (ENST00000619186)	chr17:7577019-7577155
TP53_Exon 9 (ENST00000269305);<br>TP53_Exon 9 (ENST00000622645);<br>TP53_Exon 8 (ENST00000617185);<br>TP53_Exon 8 (ENST00000610292);<br>TP53_Exon 9 (ENST00000455263);<br>TP53_Exon 5 (ENST00000510385);<br>TP53_Exon 5 (ENST00000610623);<br>TP53_Exon 5 (ENST00000619186)	chr17:7576853-7576926
TP53_Exon 10 (ENST00000622645);<br>TP53_Exon 9 (ENST00000617185);<br>TP53_Exon 6 (ENST00000510385)	chr17:7576625-7576657
TP53_Exon 10 (ENST00000455263);<br>TP53_Exon 6 (ENST00000610623)	chr17:7576537-7576584
TP53_Exon 10 (ENST00000269305);<br>TP53_Exon 9 (ENST00000610292);<br>TP53_Exon 6 (NM_001126115)	chr17:7573927-7574033
TP53_Exon 11 (ENST00000269305);<br>TP53_Exon 10 (ENST00000610292);<br>TP53_Exon 7 (NM_001126115)	chr17:7572927-7573008
