# Supplementary material for: Analysis of genetic variants in myeloproliferative neoplasms using a 22-gene next-generation sequencing panel
Source: BMC Med Genomics. 2022 Jan 15;15:10. doi: 10.1186/s12920-021-01145-0 (PMC8760696; doi:10.1186/s12920-021-01145-0)
Supplement: Supplementary file 2 — Additional file 2. Table S2. Amplicon regions. [file 12920_2021_1145_MOESM2_ESM.pdf]

Additional file 2: Table S2. Amplicon regions.

No.	Amplicon	Chromosome	Start Position	End Position
1	AMPL98763	chr9	5069847	5070002
2	AMPL98764	chr9	5069942	5070209
3	AMPL200312	chr9	5072428	5072691
4	AMPL90247	chr9	5073605	5073878
5	AMPL90231	chr9	5077317	5077554
6	AMPL90232	chr9	5077465	5077699
7	AMPL90230	chr9	5078234	5078508
8	AMPL1554	chr17	7572828	7573106
9	AMPL1120	chr17	7573853	7574119
10	AMPL1079631	chr17	7576470	7576652
11	AMPL89085	chr17	7576534	7576785
12	AMPL89078	chr17	7576758	7577034
13	AMPL89076	chr17	7576947	7577221
14	AMPL1108	chr17	7577373	7577640
15	AMPL89077	chr17	7578062	7578321
16	AMPL1155	chr17	7578184	7578446
17	AMPL1156	chr17	7578360	7578579
18	AMPL1582	chr17	7579263	7579513
19	AMPL89075	chr17	7579379	7579615
20	AMPL89084	chr17	7579585	7579843
21	AMPL89079	chr17	7579713	7579991
22	AMPL200421	chr19	13054318	13054595
23	AMPL200422	chr19	13054523	13054702
24	AMPL117175	chr19	13054634	13054811
25	AMPL89344	chr2	25457023	25457296
26	AMPL89345	chr2	25457241	25457463
27	AMPL89341	chr2	25458476	25458754
28	AMPL89342	chr2	25459665	25459930
29	AMPL89335	chr2	25461854	25462128
30	AMPL89351	chr2	25463078	25463354
31	AMPL200683	chr2	25463420	25463689
32	AMPL89336	chr2	25464282	25464554
33	AMPL89337	chr2	25464411	25464625
34	AMPL89328	chr2	25466602	25466882
35	AMPL89329	chr2	25466820	25467067
36	AMPL89330	chr2	25467017	25467290
37	AMPL89349	chr2	25467257	25467528
38	AMPL89350	chr2	25467455	25467732
39	AMPL89347	chr2	25467912	25468183
40	AMPL89348	chr2	25468123	25468398
41	AMPL89331	chr2	25468791	25469066
42	AMPL89332	chr2	25469006	25469224
43	AMPL89323	chr2	25469290	25469565
44	AMPL89324	chr2	25469472	25469740
45	AMPL89338	chr2	25469854	25470085
46	AMPL89346	chr2	25470399	25470668
47	AMPL89325	chr2	25470872	25471124
48	AMPL89326	chr2	25471065	25471217

Additional file 2: Table S2. *Cont.*

49	AMPL89334	chr2	25472420	25472700
50	AMPL89327	chr2	25474886	25475152
51	AMPL89318	chr2	25497733	25497996
52	AMPL89340	chr2	25498240	25498480
53	AMPL89320	chr2	25505121	25505378
54	AMPL89321	chr2	25505241	25505511
55	AMPL89322	chr2	25505350	25505605
56	AMPL89339	chr2	25522980	25523258
57	AMPL89343	chr2	25536682	25536954
58	AMPL89906	chr13	28592520	28592795
59	AMPL89919	chr13	28607935	28608208
60	AMPL89900	chr13	28608133	28608408
61	AMPL89896	chr13	28608365	28608627
62	AMPL90402	chr20	31019319	31019552
63	AMPL90410	chr20	31020612	31020881
64	AMPL90406	chr20	31021053	31021328
65	AMPL90407	chr20	31021257	31021526
66	AMPL90408	chr20	31021466	31021619
67	AMPL90409	chr20	31021559	31021746
68	AMPL90412	chr20	31022147	31022420
69	AMPL90413	chr20	31022344	31022616
70	AMPL90414	chr20	31022561	31022729
71	AMPL90415	chr20	31022669	31022875
72	AMPL90416	chr20	31022815	31022995
73	AMPL90417	chr20	31022935	31023187
74	AMPL90418	chr20	31023127	31023404
75	AMPL90419	chr20	31023344	31023610
76	AMPL90420	chr20	31023550	31023788
77	AMPL90421	chr20	31023727	31023979
78	AMPL90422	chr20	31023918	31024184
79	AMPL90423	chr20	31024124	31024350
80	AMPL90424	chr20	31024290	31024556
81	AMPL90425	chr20	31024496	31024667
82	AMPL90426	chr20	31024607	31024872
83	AMPL90427	chr20	31024812	31024992
84	AMPL90428	chr20	31024932	31025133
85	AMPL90429	chr20	31025073	31025256
86	AMPL32886	chr19	33792123	33792403
87	AMPL32888	chr19	33792342	33792490
88	AMPL88426	chr19	33792429	33792714
89	AMPL88427	chr19	33792654	33792858
90	AMPL88428	chr19	33792798	33793017
91	AMPL88429	chr19	33792823	33793091
92	AMPL32897	chr19	33792987	33793139
93	AMPL32898	chr19	33793079	33793347
94	AMPL317545	chr21	36164263	36164539
95	AMPL89657	chr21	36164427	36164674
96	AMPL89658	chr21	36164612	36164785
97	AMPL89659	chr21	36164644	36164907

Additional file 2: Table S2. *Cont.*

98	AMPL89660	chr21	36164761	36164944
99	AMPL89671	chr21	36171566	36171835
100	AMPL89664	chr21	36193836	36194110
101	AMPL89665	chr21	36206681	36206910
102	AMPL89666	chr21	36206860	36206985
103	AMPL89670	chr21	36231691	36231961
104	AMPL89674	chr21	36252796	36253068
105	AMPL317555	chr21	36258905	36259189
106	AMPL89662	chr21	36259126	36259402
107	AMPL89663	chr21	36259317	36259451
108	AMPL89668	chr21	36264989	36265262
109	AMPL89669	chr21	36265211	36265363
110	AMPL89667	chr21	36421010	36421288
111	AMPL89573	chr1	36931628	36931886
112	AMPL89551	chr1	36931865	36932118
113	AMPL89552	chr1	36932013	36932236
114	AMPL89553	chr1	36932149	36932377
115	AMPL89554	chr1	36932316	36932540
116	AMPL89564	chr1	36932754	36933031
117	AMPL89567	chr1	36933053	36933305
118	AMPL89559	chr1	36933339	36933610
119	AMPL89571	chr1	36936943	36937102
120	AMPL89572	chr1	36937030	36937303
121	AMPL117202	chr1	43814902	43815103
122	AMPL89822	chr21	44514676	44514947
123	AMPL89820	chr21	44524311	44524583
124	AMPL115670	chr4	55140953	55141196
125	AMPL200888	chr4	55144003	55144234
126	AMPL115657	chr4	55151945	55152194
127	AMPL90096	chr4	55561523	55561789
128	AMPL90097	chr4	55561729	55561972
129	AMPL90098	chr4	55589666	55589931
130	AMPL90095	chr4	55591996	55592269
131	AMPL90107	chr4	55593313	55593571
132	AMPL201076	chr4	55593508	55593783
133	AMPL90106	chr4	55594111	55594375
134	AMPL253513	chr4	55599207	55599388
135	AMPL90108	chr4	55602581	55602852
136	AMPL89847	chr17	74732798	74733072
137	AMPL89848	chr17	74733010	74733262
138	AMPL89849	chr17	74733193	74733461
139	AMPL1123	chr15	90631710	90631979
140	AMPL1124	chr15	90631919	90632092
141	AMPL89402	chr4	106154875	106155149
142	AMPL89403	chr4	106155089	106155280
143	AMPL89404	chr4	106155220	106155475
144	AMPL89405	chr4	106155415	106155683
145	AMPL89406	chr4	106155623	106155878
146	AMPL89407	chr4	106155818	106156067

Additional file 2: Table S2. *Cont.*

147	AMPL89408	chr4	106156007	106156279
148	AMPL89409	chr4	106156219	106156470
149	AMPL89410	chr4	106156410	106156676
150	AMPL89411	chr4	106156616	106156887
151	AMPL89412	chr4	106156827	106157102
152	AMPL89413	chr4	106157042	106157315
153	AMPL89414	chr4	106157254	106157528
154	AMPL89415	chr4	106157468	106157732
155	AMPL89416	chr4	106157672	106157951
156	AMPL89417	chr4	106157889	106158132
157	AMPL89418	chr4	106158072	106158283
158	AMPL89419	chr4	106158223	106158438
159	AMPL89420	chr4	106158378	106158622
160	AMPL89421	chr4	106162393	106162664
161	AMPL89401	chr4	106163889	106164153
162	AMPL89399	chr4	106164648	106164878
163	AMPL89400	chr4	106164816	106165005
164	AMPL1398	chr4	106180619	106180878
165	AMPL1399	chr4	106180818	106181052
166	AMPL1233	chr4	106182796	106183047
167	AMPL1414	chr4	106190693	106190957
168	AMPL1457	chr4	106193664	106193919
169	AMPL1458	chr4	106193859	106194113
170	AMPL1558	chr4	106196160	106196409
171	AMPL1559	chr4	106196349	106196621
172	AMPL1560	chr4	106196560	106196821
173	AMPL1561	chr4	106196761	106197034
174	AMPL1562	chr4	106196974	106197249
175	AMPL1563	chr4	106197189	106197407
176	AMPL1564	chr4	106197347	106197501
177	AMPL1565	chr4	106197441	106197709
178	AMPL47323	chr11	119148794	119149059
179	AMPL200758	chr11	119149187	119149457
180	AMPL253478	chr9	133738047	133738317
181	AMPL200757	chr9	133738257	133738510
182	AMPL200561	chr9	133747442	133747711
183	AMPL200414	chr9	133748144	133748418
184	AMPL200415	chr9	133748358	133748545
185	AMPL200672	chr9	133750218	133750480
186	AMPL200554	chr9	133753746	133754011
187	AMPL253505	chr9	133755364	133755636
188	AMPL90657	chr7	148504633	148504900
189	AMPL90666	chr7	148506075	148506342
190	AMPL317126	chr7	148506310	148506575
191	AMPL253481	chr7	148507328	148507604
192	AMPL1138	chr7	148508594	148508869
193	AMPL253507	chr7	148511012	148511272
194	AMPL90663	chr7	148511912	148512187
195	AMPL253486	chr7	148512501	148512736

Additional file 2: Table S2. *Cont.*

196	AMPL253496	chr7	148513689	148513958
197	AMPL90660	chr7	148514249	148514516
198	AMPL90668	chr7	148514757	148515022
199	AMPL90669	chr7	148514962	148515234
200	AMPL90658	chr7	148516593	148516867
201	AMPL90670	chr7	148523365	148523600
202	AMPL90671	chr7	148523540	148523803
203	AMPL90672	chr7	148524083	148524338
204	AMPL90673	chr7	148524278	148524514
205	AMPL90674	chr7	148525756	148526022
206	AMPL90675	chr7	148526789	148527039
207	AMPL90667	chr7	148529637	148529905
208	AMPL90661	chr7	148543458	148543731
209	AMPL90655	chr7	148544118	148544334
210	AMPL90656	chr7	148544274	148544448
211	AMPL417935	chr5	170837379	170837627
212	AMPL1449	chr2	198266297	198266560
213	AMPL90214	chr2	198266483	198266751
214	AMPL1451	chr2	198266691	198266880
215	AMPL1453	chr2	198267125	198267374
216	AMPL1454	chr2	198267314	198267578
217	AMPL90202	chr2	198267560	198267821
218	AMPL90132	chr2	209113057	209113312
219	AMPL90133	chr2	209113252	209113426
