# Supplementary material for: Analysis of genetic variants in myeloproliferative neoplasms using a 22-gene next-generation sequencing panel
Source: BMC Med Genomics. 2022 Jan 15;15:10. doi: 10.1186/s12920-021-01145-0 (PMC8760696; doi:10.1186/s12920-021-01145-0)
Supplement: Supplementary file 3 — Additional file 3. Table S3. Details of variants present in reference standards. [file 12920_2021_1145_MOESM3_ESM.pdf]

Additional file 3: Table S3. Details of variants present in reference standards.

Reference standard	Gene	Variant	Expected/Target Allelic Frequency, %
Horizon Tru-Q 0 (Wildtype standard)	*EGFR*	G719S	16.70
	BRAF	V600E	8.00
	KRAS	G13D	25.00
	PIK3CA	H1047R	30.00
Horizon Tru-Q 1	*BRAF*	V600E	8.00
	BRAF	V600K	4.00
	EGFR	G719S	16.70
	EGFR	T790M	4.20
	FLT3	ΔI836	5.00
	IDH1	R132C	5.00
	JAK2	V617F	5.00
	KRAS	G12A	5.00
	KRAS	G12R	5.00
	KRAS	G13D	25.00
	MEK1	P124L	5.00
	NOTCH1	L1600P	4.80
	NRAS	Q61K	5.00
	PIK3CA	H1047R	30.00
Horizon Tru-Q 7	*ABL1*	T315I	1.30
	ALK	F1174L	1.30
	BRAF	V600E	8.00
	BRAF	V600G	1.00
	BRAF	V600K	1.00
	BRAF	V600M	1.00
	BRAF	V600R	1.00
	EGFR	G719S	16.70
	EGFR	L858R	1.00
	EGFR	L861Q	1.00
	EGFR	T790M	1.00
	EGFR	ΔE746-A750	1.00
	FGFR2	S252W	1.00
	FLT3	D835Y	1.30
	FLT3	ΔI836	1.30
	GNA11	Q209L	1.30
	GNAQ	Q209L	1.30
	IDH1	R132C	1.30
	IDH1	R132H	1.30
	IDH2	R140Q	1.30
	IDH2	R172K	1.30
	JAK2	V617F	1.30
	KIT	D816V	1.30
	KRAS	A146T	1.30
	KRAS	G12A	1.30
	KRAS	G12C	1.30
	KRAS	G12D	1.30
	KRAS	G12R	1.30
	KRAS	G12S	1.30
	KRAS	G12V	1.30
	KRAS	G13D	25.00

Additional file 3: Table S3. *Cont.*

Reference standard	Gene	Variant	Expected/Target Allelic Frequency, %
Horizon Tru-Q 7	*KRAS*	Q61H	1.30
	KRAS	Q61L	1.30
	MEK1	P124L	1.30
	MET	Y1253D	1.00
	NOTCH	L1601P	1.30
	NRAS	Q61H	1.30
	NRAS	Q61K	1.30
	NRAS	Q61L	1.30
	NRAS	Q61R	1.30
	PDGFRA	D842V	1.30
	PIK3CA	E542K	1.30
	PIK3CA	E545K	1.30
	PIK3CA	H1047R	30.00
Seraseq Myeloid Mutation DNA Mix	*CALR*	L367fs*46	5.00
	CSF3R	T618I	5.00
	FLT3	dup c.1759_1800	5.00
	IDH1	R132C	5.00
	JAK2	V617F	5.00
	MPL	W515L	5.00
	NPM1	W288fs*12	5.00
	SF3B1	K700E	5.00
	SF3B1	K666N	5.00
	SRSF2	P95_R102del	5.00
	ABL1	T315I	10.00
	ASXL1	E635fs*15	10.00
	ASXL1	G646fs*12	10.00
	BRAF	V600E	10.00
	CBL	L380P	10.00
	CBL	R420Q	10.00
	FLT3	dup chr 13:28,608,250-28,608,277	10.00
	FLT3	D835Y	10.00
	JAK2	N542_E543del	10.00
	MYD88	L265P	10.00
	U2AF1	S34F	10.00
	CEBPA	H24fs*84	15.00
	CEBPA	K313_V314insK	15.00
