# Supplementary material for: Analysis of genetic variants in myeloproliferative neoplasms using a 22-gene next-generation sequencing panel
Source: BMC Med Genomics. 2022 Jan 15;15:10. doi: 10.1186/s12920-021-01145-0 (PMC8760696; doi:10.1186/s12920-021-01145-0)
Supplement: Supplementary file 4 — Additional file 4. Table S4. Primers used in variant confirmation. [file 12920_2021_1145_MOESM4_ESM.pdf]

Additional file 4: Table S4. Primers used in variant confirmation.

Target	RefSeq	Primer sequence (5' to 3')	Product size (bp)
ABL1 exon 6 p.N331S	NM_005157	F: CTGAGGAGCAGAGTCAGAATCC R: CACTGAGGTTAGAAGCTGCG	397
ASXL1 exon 12 p.Y591fs*0	NM_015338	F: GTGGCATATAACAGCCCTTGAG R: ACCTCCCTCATCGGTGGC	462
ASXL1 exon 12 p.G646Wfs*10	NM_015338	F: TTACCAGATATGCCCCCGGA R: AGCTCTGGACATGGCAGTTC	389
ASXL1 exon 12 p.L731Yfs*12	NM_015338	F: GAGCACCCCTGGAAAGTGTA R: TGGTGCCAGACTCACATTCA	350
ASXL1 exon 12 p.Q1433Q	NM_015338	F: GGGCATAGTCCCCTGGAACT R: GGCTCGCACCAAAGTTTGCT	286
CALR exon 9 p.K385T; p.K385Ifs*46/ p.L367Tfs*45	NM_004343	F: GAGTTTGGCAACGAGACGTG R: CCCAAATCCGAACCAGCCT	440/394
DNMT3A exon 10 p.P385P	NM_175629	F: ATAAAGGACAGAAGATTCGGC R: TTCTCTTCTGGGTGCTGATA	229
JAK2 exon 14 p.V617F	NM_004972	F: CTTTCTTTGAAGCAGCAAGT R: AGTTTACACTGACACCTAGC	189
RUNX1 exon 6 p.Q308H	NM_001001890	F: TAGGCGGTATCATCCTGGGTAGC R: TCAGTAGGGCCTCCACACGGC	577
SF3B1 exon 15 p.K700E	NM_012433	F: TTCTGCAGTTTGGCTGAATAGT R: ATAGCCTTCAAGAAAGCAGCC	372
TET2 exon 3 p.A304V	NM_001127208	F: CATTGCGGTGCAGAAAACCA R: AGTGAACACTGAGCTTTGCT	448
TET2 exon 3 p.F868L	NM_001127208	F: TGTCCAAATGGGACTGGAGG R: GATGCCACCTTAGAGCAGCA	464
TET2 exon 5 p.I1195V	NM_001127208	F: GGCATGAGTCTTTGATCTGGG R: CCGTTCATTTCTCAGGATGTGG	315
TET2 exon 6 p.Y1245C	NM_001127208	F: TGCCCTAATTGTGATCTAAACATGA R: ATGTAAAAGTGCACGCTGAA	431
TET2 exon 7 p.D1314Mfs*48	NM_001127208	F: ACAGAGTTAGATTAGACTTCTTTT R: ACAGTTTGGGAAAAACTTTGAT	390
TET2 exon 11 p.E1513G	NM_001127208	F: TCAGAACCCTGACTTTGCTCTT R: GGGCTGGTGCTTCCATAGAT	398
U2AF1 exon 6 p.Q157P	NM_006758	F: ATTCCACCTTACTATACCTGAGTGT R: CATGAAGTTGCAGAAGCCGC	373
