# Supplementary material for: Analysis of genetic variants in myeloproliferative neoplasms using a 22-gene next-generation sequencing panel
Source: BMC Med Genomics. 2022 Jan 15;15:10. doi: 10.1186/s12920-021-01145-0 (PMC8760696; doi:10.1186/s12920-021-01145-0)
Supplement: Supplementary file 5 — Additional file 5. Fig. S1. Representative image of (a) Labchip gel and (b) multiple overlay electropherogram of the constructed libraries of the reference standards. The image shows the quality and quantity of input DNA for the second NGS run for the technical validation of the custom NGS panel. The majority of the constructed libraries were within the targeted size range of 400 bp. [file 12920_2021_1145_MOESM5_ESM.pdf]

(a)

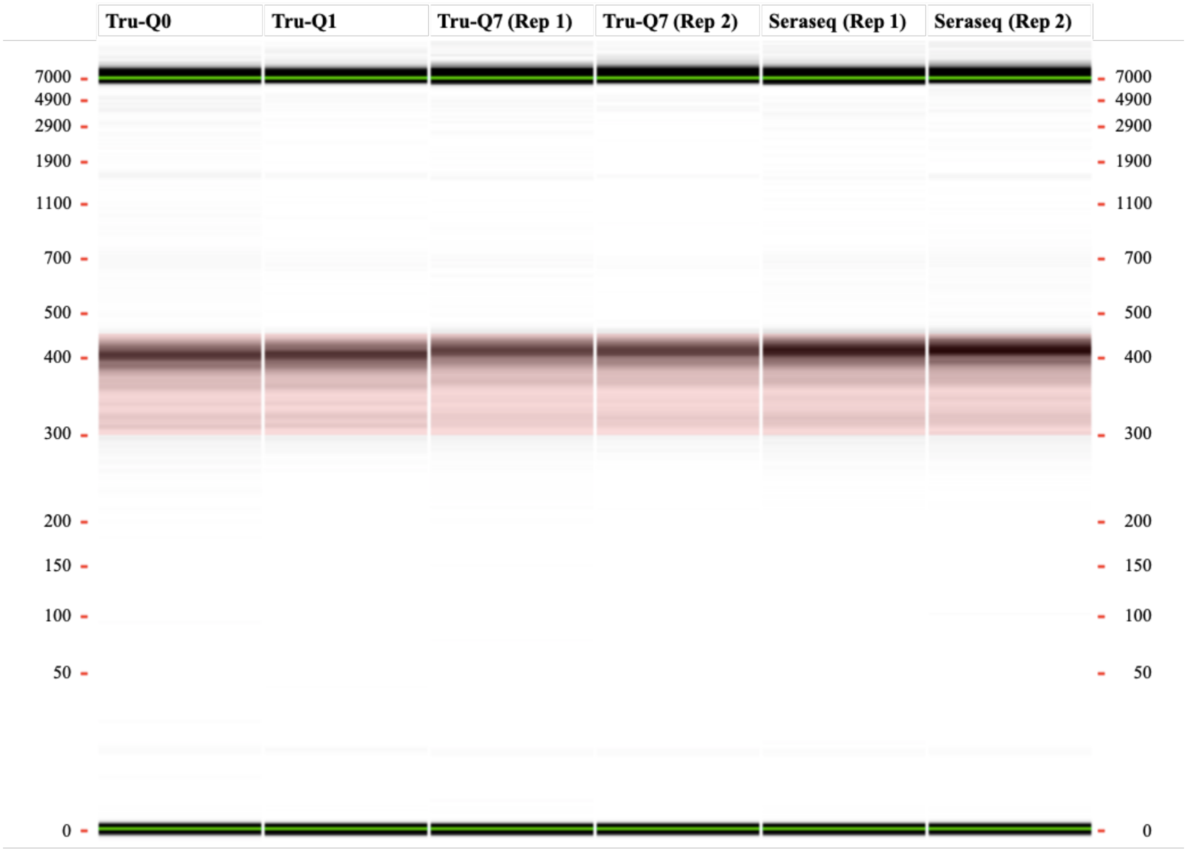

(b)

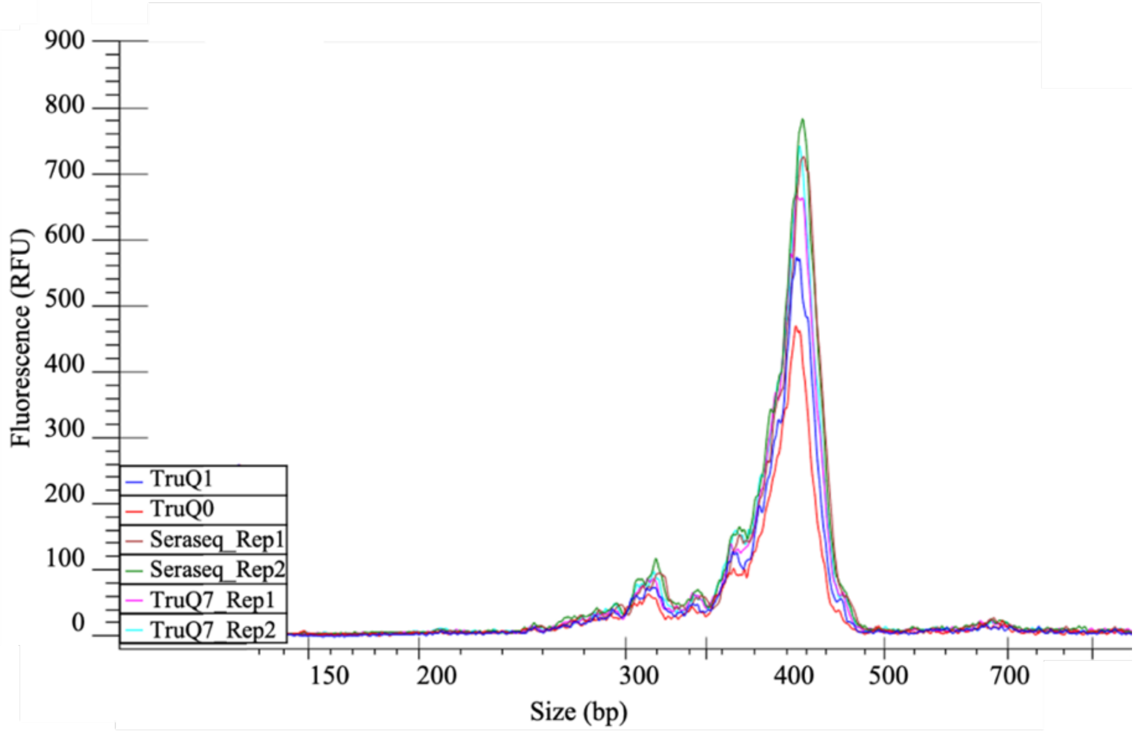

Additional file 5: Fig. S1. Representative image of (**a**) Labchip gel and (**b**) multiple overlay electropherogram of the constructed libraries of the reference standards. The image shows the quality and quantity of input DNA for the second NGS run for the technical validation of the custom NGS panel. The majority of the constructed libraries were within the targeted size range of 400 bp.
