# Supplementary material for: Analysis of genetic variants in myeloproliferative neoplasms using a 22-gene next-generation sequencing panel
Source: BMC Med Genomics. 2022 Jan 15;15:10. doi: 10.1186/s12920-021-01145-0 (PMC8760696; doi:10.1186/s12920-021-01145-0)
Supplement: Supplementary file 6 — Additional file 6. Table S5. Sequencing metrics of NGS runs for the technical validation of the custom NGS panel. [file 12920_2021_1145_MOESM6_ESM.pdf]

Additional file 6: Table S5. Sequencing metrics of NGS runs for the technical validation of the custom NGS panel.

	**Forward read**		**Reverse read**	
**Metric**	**Run 1**	**Run 2**	**Run 1**	**Run 2**
Cluster Density (K/mm2)	860 ± 28	878 ± 17	860 ± 28	878 ± 17
Clusters PF (%)[1]	94.76 ± 0.53	93.09 ± 1.44	94.76 ± 0.53	93.09 ± 1.44
% >= Q30[2]	97.80	97.35	96.12	95.27

[1] The percentage of clusters passing filter (%PF) is an indication of signal purity from each cluster. [2] Q30 indicates an accuracy of 99.9%. The percentage of bases >Q30 is averaged across the entire run. The Miseq System specifications are 865-965 K/mm2 cluster density and >80% of bases above Q30. K, kilobases; PF, passing filter.
