# Supplementary material for: Analysis of genetic variants in myeloproliferative neoplasms using a 22-gene next-generation sequencing panel
Source: BMC Med Genomics. 2022 Jan 15;15:10. doi: 10.1186/s12920-021-01145-0 (PMC8760696; doi:10.1186/s12920-021-01145-0)
Supplement: Supplementary file 7 — Additional file 7. Table S6. Amplicon coverage data for the technical validation of the custom NGS panel. [file 12920_2021_1145_MOESM7_ESM.pdf]

Additional file 7: Table S6. Amplicon coverage data for the technical validation of the custom NGS panel.

Sample	Amplicon mean coverage depth		Uniformity of coverage[3]	
	Run 1	Run 2	Run 1	Run 2
Tru-Q0	6261	4643	98.17	98.63
Tru-Q1	4589	6455	98.17	98.17
Tru-Q7-rep1	6887	6304	98.63	98.63
Tru-Q7-rep2	4646	6717	98.17	98.17
Seraseq-rep1	7944	6256	98.17	98.17
Seraseq-rep2	6543	6321	98.17	98.63

[3] The uniformity of coverage is measured as the percentage of targeted base positions in which the read depth is 20% greater than that of the mean region target coverage depth.
