# Supplementary material for: Analysis of genetic variants in myeloproliferative neoplasms using a 22-gene next-generation sequencing panel
Source: BMC Med Genomics. 2022 Jan 15;15:10. doi: 10.1186/s12920-021-01145-0 (PMC8760696; doi:10.1186/s12920-021-01145-0)
Supplement: Supplementary file 8 — Additional file 8. Fig. S2. Amplicon coverage across reference standards in the technical validation of the custom NGS panel. More details on the amplicon regions can be found in Additional file 2: Table S2 and Additional file 10. [file 12920_2021_1145_MOESM8_ESM.pdf]

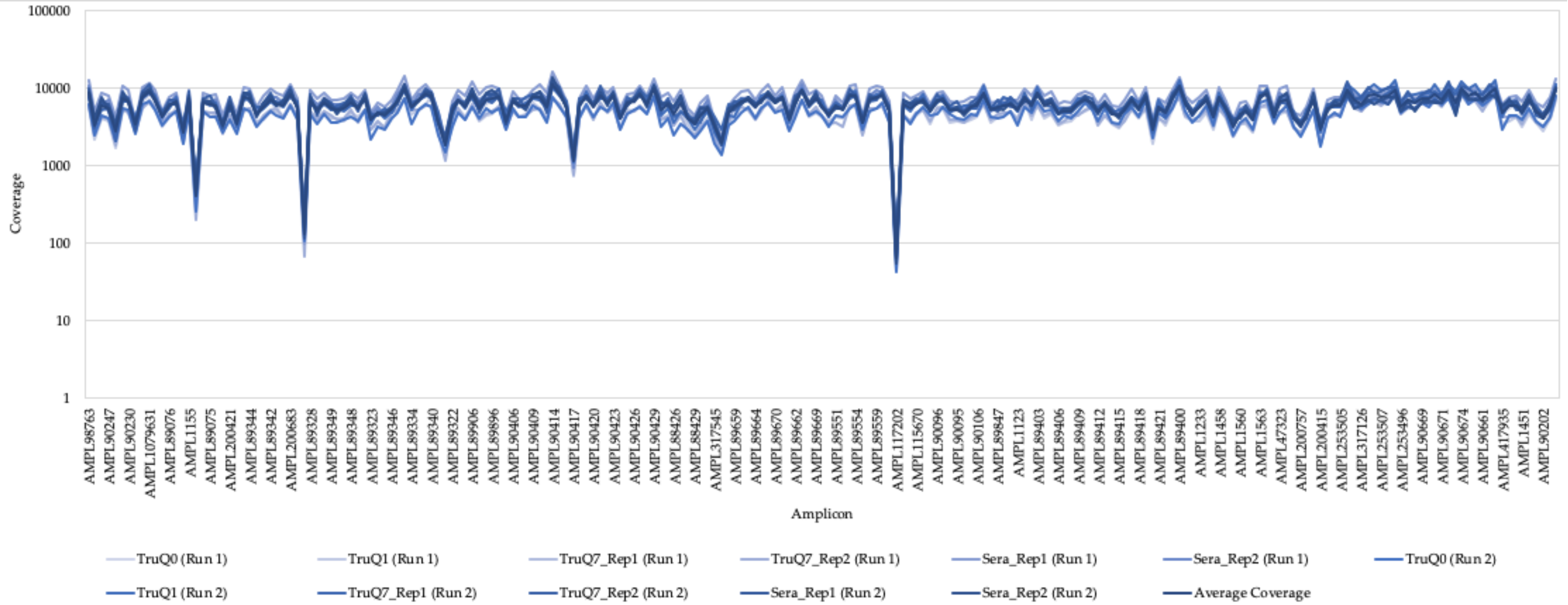

Additional file 8: Fig. S2. Amplicon coverage across reference standards in the technical validation of the custom NGS panel. More details on the amplicon regions can be found in Additional file 2: Table S2 and Additional File 10.
