# Supplementary figures and images for: Analysis of genetic variants in myeloproliferative neoplasms using a 22-gene next-generation sequencing panel
Source: BMC Med Genomics. 2022 Jan 15;15:10. doi: 10.1186/s12920-021-01145-0 (PMC8760696; doi:10.1186/s12920-021-01145-0)

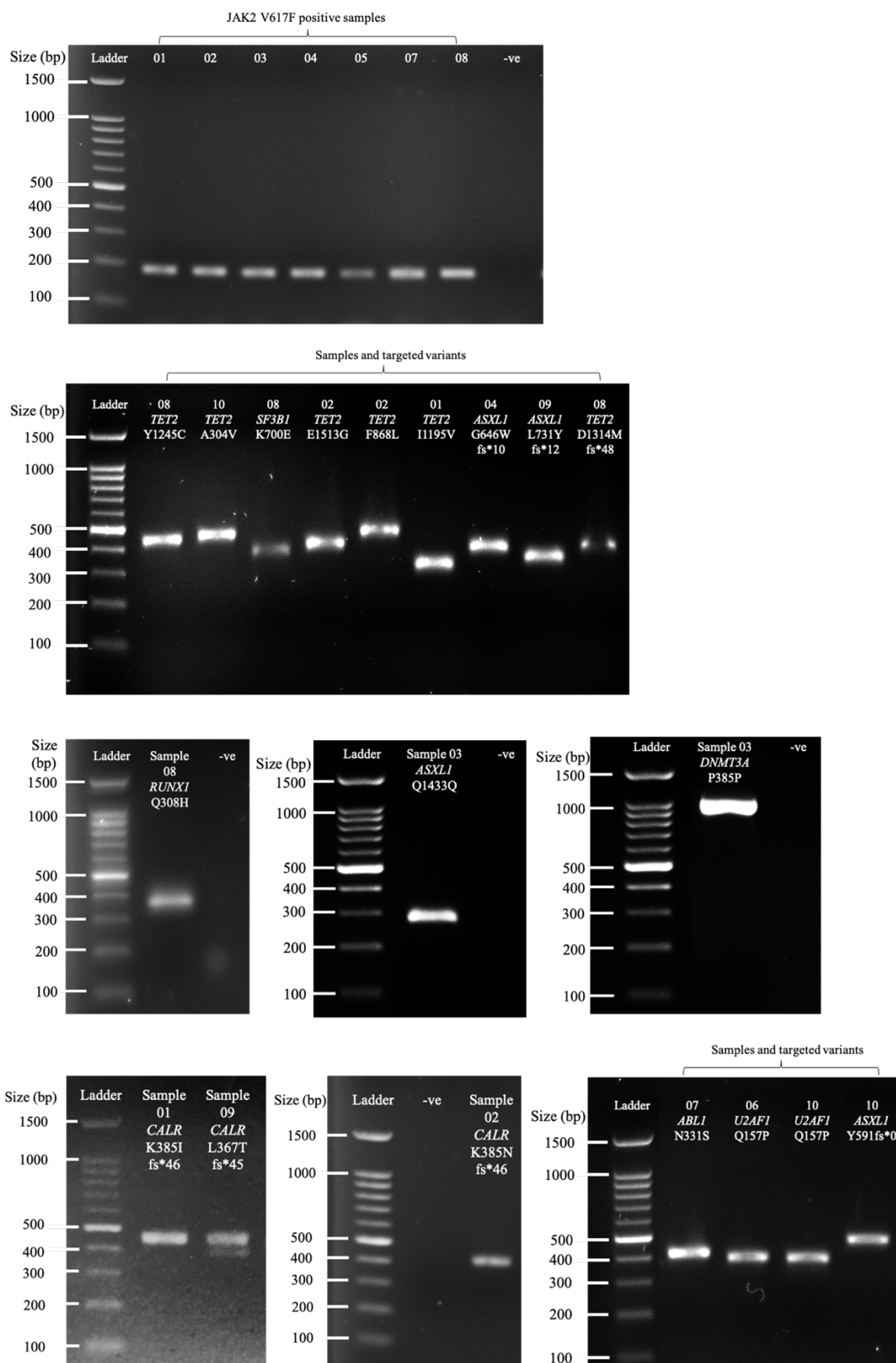

Additional file 9: Fig. S3. Gel electrophoresis images. -ve, Negative control.

Supplement: Supplementary file 9 — Additional file 9. Fig. S3. Gel electrophoresis images. [file 12920_2021_1145_MOESM9_ESM.pdf]
