# Supplementary material for: Analysis of genetic variants in myeloproliferative neoplasms using a 22-gene next-generation sequencing panel
Source: BMC Med Genomics. 2022 Jan 15;15:10. doi: 10.1186/s12920-021-01145-0 (PMC8760696; doi:10.1186/s12920-021-01145-0)
Supplement: Supplementary file 11 — Additional file 11. Fig. S4. Distribution of GC content across AMPL117202 (MPL exon 10, chr1:43814902-43815103). [file 12920_2021_1145_MOESM11_ESM.pdf]

```
>NC_000001.10:43814902-43815103 Homo sapiens chromosome 1,
GRCh37.p13 Primary Assembly
TGGGCCGAAGTCTGACCCTTTTTGTCTCCTAGCCTGGATCTCCTTGGTGACCGCTCTGCAT
CTAGTGCTGGGCCTCAGCGCCGTCCTGGGCCTGCTGCTGCTGAGGTGGCAGTTTCCTGCAC
ACTACAGGTACCGCCCCCGCCAGGCAGGAGACTGGCGGTGGACCAGGTGGAGCCGAAGGCC
TGTAAACAGGCATTCTTGG
```

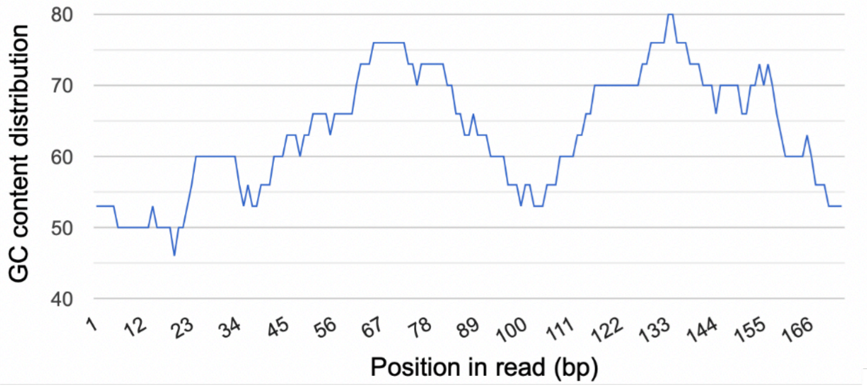

Additional file 11: Fig. S4. Distribution of GC content across AMPL117202 (*MPL* exon 10, chr1:43814902-43815103). Graph generated using the GC Content Calculator by Guerra et al., 2016.

Reference

Guerra ÁP, Calvo EP, Wasserman M, Chaparro-Olaya J. Producción de proteínas recombinantes de Plasmodium falciparum en Escherichia coli. Biomédica. 2016;36(Sup1):97-108.
